# Mouse and human striatal projection neurons compared - *somatodendritic arbor, spines and in silico analyses*

**DOI:** 10.1371/journal.pcbi.1013569

**Published:** 2025-10-09

**Authors:** Alexander Kozlov, Lidia Blazquez-Llorca, Ruth Benavides-Piccione, Asta Kastanauskaite, Ana I. Rojo, Alberto Muñoz, Antonio Cuadrado, Javier DeFelipe, Sten Grillner

**Affiliations:** 1 Department of Neuroscience, Karolinska Institutet, Stockholm, Sweden; 2 Science for Life Laboratory, Division of Computational Science and Technology, School of Electrical Engineering and Computer Science (EECS), KTH Royal Institute of Technology, Stockholm, Sweden; 3 Laboratorio Cajal de Circuitos Corticales, Centro de Tecnología Biomédica (CTB), Universidad Politécnica de Madrid (UPM), Madrid, Spain; 4 Centro de Investigación Biomédica en Red sobre Enfermedades Neurodegenerativas (CIBERNED), ISCIII, Madrid, Spain; 5 Instituto Cajal, Consejo Superior de Investigaciones Científicas (CSIC), Madrid, Spain; 6 Department of Biochemistry, Faculty of Medicine, Autonomous University of Madrid (UAM), Madrid, Spain. Instituto de Investigaciones Biomédicas Sols-Morreale (CSIC-UAM), Madrid, Spain; 7 Instituto de Investigación Sanitaria La Paz (IdiPaz), Madrid, Spain; 8 Departamento de Biología Celular, Facultad de Ciencias Biológicas, Universidad Complutense de Madrid (UCM), Madrid, Spain; The University of Iowa College of Engineering, UNITED STATES OF AMERICA

## Abstract

Dysfunction of the basal ganglia is implicated in a wide range of neurological and psychiatric disorders. Our understanding of the operation of the basal ganglia is largely derived on data from studies conducted on mice, which are frequently used as model organisms for various clinical conditions. The striatum, the largest compartment of the basal ganglia, consists of 90–95% striatal projection neurons (SPNs). It is therefore crucial to establish if human and mouse SPNs have distinct or similar properties, as this has implications for the relevance of mouse models for understanding the human striatum. To address this, we compared the general organization of the somato-dendritic tree of SPNs, the dimensions of the dendrites, the density and size of spines (spine surface area), and ion channel subtypes in human and mouse SPNs. Our findings reveal that human SPNs are significantly larger, but otherwise the organisation of the dendritic tree (dendrogram) with an average of approximately 5 primary dendrites, is similar in both species. Additionally in both humans and mice, over 90% of the spines are located on the terminal branches of each dendrite. Human spines are somewhat larger (4.3 versus 3.1 μm^2^) and the terminal dendrites have a uniform diameter in both humans and mice, although somewhat broader in the latter (1.0 versus 0.6 μm). The composition of ion channels is also largely conserved. These data have been used to simulate human SPNs building on our previous detailed simulation of mouse SPNs. We conclude that the human SPNs essentially appear as enlarged versions of the mouse SPNs. This similarity suggests that both species process information in a comparable manner, supporting the relevance of mouse models for studying the human striatum.

## Author summary

The basal ganglia, a brain region critical for movement and behavior, are implicated in many neurological and psychiatric disorders. Much of what we know about this system comes from studies in mice, so it’s essential to understand how well mouse models reflect human brain structure. We focused on striatal projection neurons (SPNs), which make up the vast majority of cells in the striatum—the largest part of the basal ganglia. By comparing human and mouse SPNs, we found that human neurons are significantly larger but share a remarkably similar structure with mouse neurons. Both species have dendrites (the branches that receive signals) organized in the same way, with most spines—the tiny protrusions on dendrites—located at the distal segments. Ion channel composition, which affects electrical signaling, is also largely conserved. These similarities suggest that mouse SPNs process information much like their human counterparts, reinforcing the value of mice in brain research.

## Introduction

The basal ganglia play a major role in motor learning and selection of behaviour, and the basic design with subnuclei, cell types and transmitters are conserved throughout vertebrate evolution [1]. Interference with the operation of the basal ganglia in humans leads to neurological disorders such as Parkinson’s disease (PD) or Huntington’s disease (HD) (DeLong 1990) or psychiatric disorders such as attention deficit hyperactivity disorder (ADHD). PD is characterised by a marked hypokinesia and conversely in HD to a hyperkinesia, testifying to the importance of the basal ganglia for the normal control of behaviour [2,3]. Central to the function of the basal ganglia is the large input structure, the striatum dominated by striatal projection neurons (SPNs; ~ 95% in rodents). The value in humans is uncertain but not lower than ~75%. They project to downstream structures within the basal ganglia [4–6]. The operation of the different types of SPNs are strongly affected in both PD and HD [2,7] and their morphology is also altered [8]. In this study, we analyse and compare the characteristics of SPNs in mice and humans by examining their somatodendritic features in detail and conducting an *in silico* analysis. Our goal is to evaluate the extent to which mouse networks can serve as models for human networks under physiological conditions, as well as to explore their relevance for basal ganglia dysfunction, such as in PD.

The SPNs are characterized by their medium size and the presence of dendritic spines (for simplicity, spines), a feature originally described by Cajal in the striatum [9,10], as well as in other parts of the brain, including the cerebral cortex and cerebellum. Due to their size and the presence of spines, the SPNs were initially referred to as medium spiny neurons, as their functional output had not yet been defined. The presence of spines is particularly significant, as they are now recognised as key elements in the organisation of synaptic connections and are thought to play a crucial role in learning, memory, and cognition [11,12].

SPNs are traditionally classified into two main subtypes [13]: (1) direct pathway SPNs (dSPNs), which express D1 dopamine receptors and project to the output nuclei—the internal globus pallidus (GPi) and substantia nigra pars reticulata (SNr); and (2) indirect pathway SPNs (iSPNs), which express D2 receptors and project to the external globus pallidus (GPe). These two major SPN types are conserved across the vertebrate lineage, from lampreys to primates [1], and are present in approximately equal proportions. A small population of SPNs co-expresses both D1 and D2 receptors but follows a projection pattern similar to that of iSPNs [14]. Beyond this classical dichotomy, SPNs exhibit remarkably similar morphology and baseline electrophysiological profiles, but they differ in the expression of numerous signaling molecules [15]. These anatomical and molecular distinctions likely underlie functional specialization within the striatum. Concerning striosomes, that represent a smaller proportion of striatum, they also contain dSPN and iSPNs, projecting to SNc and GPe respectively. They have in the mouse similar but not identical properties to those in the matrix area. However, as our study analyzed a relatively limited number of cells and did not systematically distinguish between these SPN subpopulations, this represents a limitation in the generalizability of our findings.

Numerous studies regarding the operation of the basal ganglia and their role in behaviour have over the last few decades been carried out with mainly mouse as the model system [16–20]), including the details of the microcircuitry with cell types, their membrane properties, types of synaptic interaction and input from the cortex and thalamus. In contrast, and for obvious reasons, the corresponding type of information is mostly missing in humans, since no patch-clamp recordings have ever been performed from human SPNs. Accounts of electrophysiological activity in the human striatum [21,22]) relate to extracellular recordings during deep brain stimulation (DBS) surgery in PD patients and provide no direct information about single-cell electrophysiology. Intracellular recordings, on the other hand, were only possible in cell cultures so far [23,24]. Although the first images of the stained human striatal neurons depicting their magnificent spine-laden dendrites appeared in Cajal’s drawings [10], their description has long remained qualitative. Limited quantitative characterisation of the dendritic morphology of human SPNs was known from the number of PD studies [25,26,8], however, no three-dimensional reconstructions enabling complete morphometric analysis has been available so far. In the current study we make a detailed comparison between mice and humans of the somatodendritic characteristics, including the spines of the SPNs. From several single cell RNA sequencing studies, we know which ion channel subtypes are expressed in both mice and humans [27–32]. This information is critical for estimating the properties of the human SPNs and their function through a detailed computational model. Given the extensive background information present in the mouse, which has been successfully used also as a model system for studying PD in humans, mostly after denervation of the dopamine supply through different mechanisms [17], but also for a variety of neurological and psychiatric disorders, we adapt our detailed computational models of mouse SPNs [33,34] to human SPNs.

Our aim here is to establish how similar or dissimilar the SPNs of mouse and humans are. We compare the general organisation of the somatodendritic tree, dimensions of the dendrites, the density and shape of spines, and the composition of ion channel subtypes in human and mouse SPNs [30,31]. Human SPNs are somewhat larger than those of the mouse, but the overall organisation of the dendritic tree (seen, e.g., in Scholl analyses and dendrograms), we show is similar. The neuronal somata and the dendritic branches have a somewhat larger diameters in humans and the total span of the dendritic field is also larger in humans, primarily due to the longer terminal dendritic segments. Spines start to appear on dendrites at around 30 μm from the soma and the largest number of spines are located at the most distal dendritic segments. Over 90% of the spines are located on the terminal dendritic sections in both species and the total number of spines is comparable due to the lower spine density observed in human neurons. The total spine area is larger in human than mouse SPNs on average, 4.3 versus 3.1 μm^2^. This detailed information on the somatodendritic properties, dimension of dendrites and the expression of the ion channels will provide the basis for modelling the human SPNs and allow a comparison of their properties with those of the detailed models of mouse SPNs developed earlier [33,34].

## Results

### The general structure of SPNs in mouse and human

**Fig 1:**
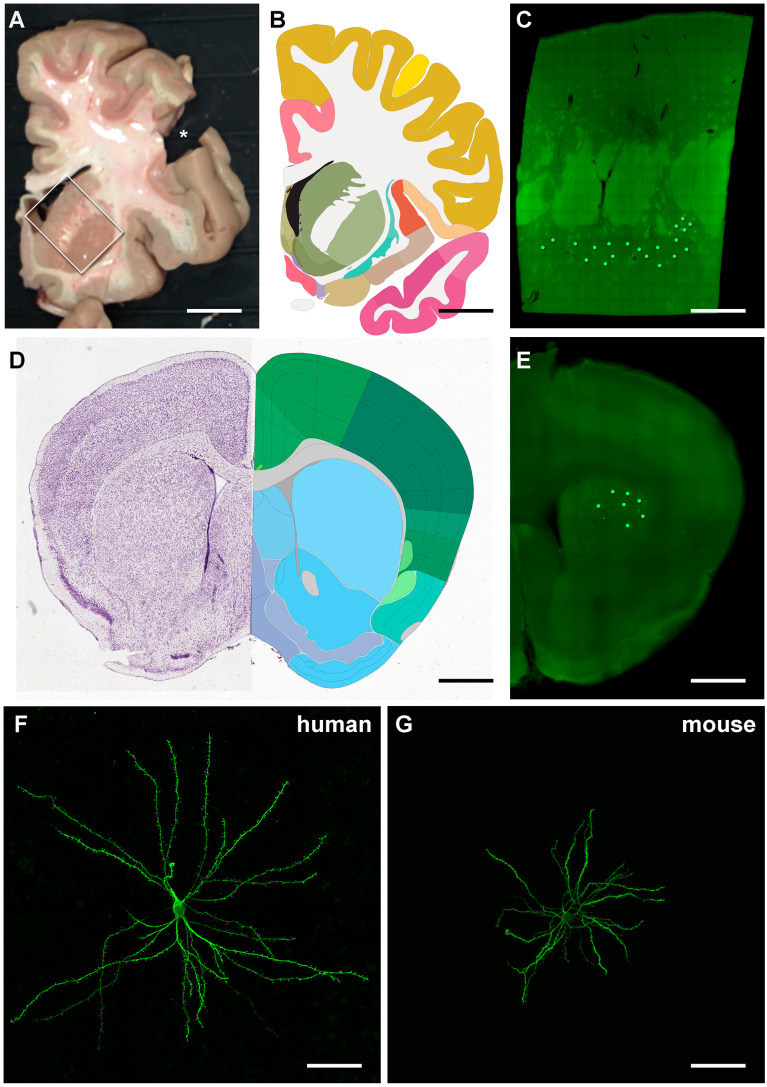
Overview and confocal photomicrographs of human and mouse striatal projection neurons (SPNs) injected with Lucifer Yellow (LY) in the human putamen and the mouse caudoputamen (CPu). **(A)** Macroscopic image of a thick coronal human brain slice. The rectangle surrounds the caudate and putamen regions. The asterisk indicates the location where brain tissue was removed for further processing and analysis. **(B)** Schematic drawing from the human brain reference atlas of the Allen Institute at a similar level of the image in **A** (https://atlas.brain-map.org/). **(C)** Image of a coronal section of the human brain containing the caudate and putamen regions. Note that the internal capsule is visible. Green dots point out the approximate location where SPNs were intracellularly injected with LY in the putamen region. **(D)** Nissl staining and schematic drawing from the mouse brain reference atlas of the Allen Institute at a similar level of the image in **E** (https://atlas.brain-map.org/). CPu is shown in light blue colour. **(E)** Coronal mouse brain section containing the CPu region. Green dots point to the approximate location where SPNs were intracellularly injected with LY in the CPu region. **(F, G)** Confocal photomicrographs showing an injected human (**F**) and mouse **(G)** SPN. Scale bar is 1.56 cm in A; 1.36 cm in B; 3 mm in C; 1.06 mm in D; 0.77 mm in E; and 66 µm in F and **G.**

In this study we compare SPNs of humans with those of mice concerning their soma and the organisation of the dendritic trees and the properties of their dendritic spines. Fig 1A-C shows the location of the human SPNs reconstructed and in Fig 1F a reconstructed SPN is shown with an extensive dendritic arbor studded with spines except for the most proximal parts. For comparison (Figs 1D, 1E, 1G and S1) a mouse SPN is illustrated, at the same magnification as the human in F. The somata of the human SPNs are medium-sized, spherical or ovaloid, 9.7 μm along the shortest dimension and 22 μm in the longest axis (the minimal and maximal Feret diameters determined from the soma outline, n = 27), mean diameter 17.4 μm (12.9 μm in mouse, minimal and maximal Feret diameters 6.5 and 16.8 μm, respectively, n = 31). The axon originates at the soma or occasionally in a proximal dendrite [35].

**Fig 2:**
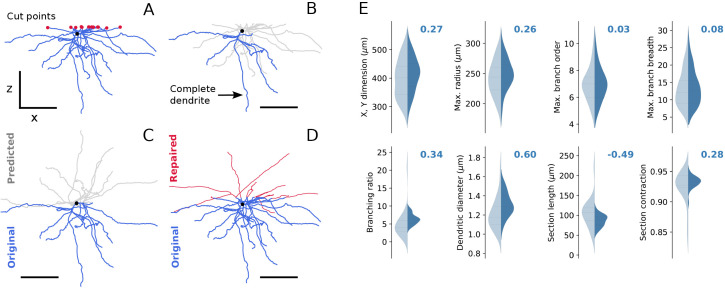
Repairing morphological reconstructions (soma centre as black dot, dendrite centre lines in blue, diameter not up to scale). **(A)** Cut points are identified in the **(x, y)**-plane as dendritic terminal nodes in the vicinity of the maximum value of the z-coordinate of the reconstruction (red dots). Scale bars are 100 μm. **(B)** Complete dendrites are selected as dendritic branches not containing cut points (shown in blue). **(C)** The predicted morphology is composed of half of the original morphology artificially cut at the level of the soma and its mirror image flipped along the z-axis (grey lines). **(D)** Incomplete dendrites are extended using parts of the complete dendrites of the same topological order as the corresponding cut points (shown in red). **(E)** Validation of the repair process for human SPNs. Distributions of morphometric features of the repaired reconstructions (dark blue, 147 dendrites in 27 reconstructions) is compared to the morphometry of the complete dendrites (light blue, 35 complete dendrites), Z-scores are shown in bold for each morphometric feature.

The SPNs were collected from thick slices of striatum and most of their dendritic arbour was included within the section (see Methods; see also S2 Fig for nomenclature of dendritic morphology). Some dendrites, however, were incomplete due to technical limitations relative to the distance from where cells were injected and were repaired before the reconstructions entered further into the modelling pipeline which relies on completeness and variability of morphological features [36]. Fig 2A illustrates a typical case, with the red dots showing the cut dendritic branches and Fig 2B shows dendrites that are intact. Complete dendrites were used in the analysis of morphometry of the dendritic branches, replacement of the sliced sections and validation of the repair process. Dendritic field of an average SPN is spherically symmetric and incomplete branches can be labelled as a set of incomplete terminal nodes in the vicinity of a single surface (cut plane). To have an estimate of the overall size of the original morphology one can, as shown in Fig 2C flip the intact half of the dendritic arbor at the soma level to add the mirror image and compute the predicted morphometry. We used portions of the complete dendrites of the same topological order as the cut branches to “repair” the incomplete dendrites and recover the cell symmetry (Fig 2D) (see also [36–38], for comparable morphology repair methods). Fig 2E (human SPNs) provides a validation of the method of transplanting dendrites (dark blue) with those of complete dendrites (light blue). Clearly the complete and repaired dendrites are similar regarding their dimensions, length, branching order etc., which indicates that the repair process provides a reliable method of estimating the entire somato-dendritic arbour of a given SPN (validation for mouse reconstructions is in S3 Fig).

### Characteristics of the dendritic tree of human and mouse SPNs

**Fig 3:**
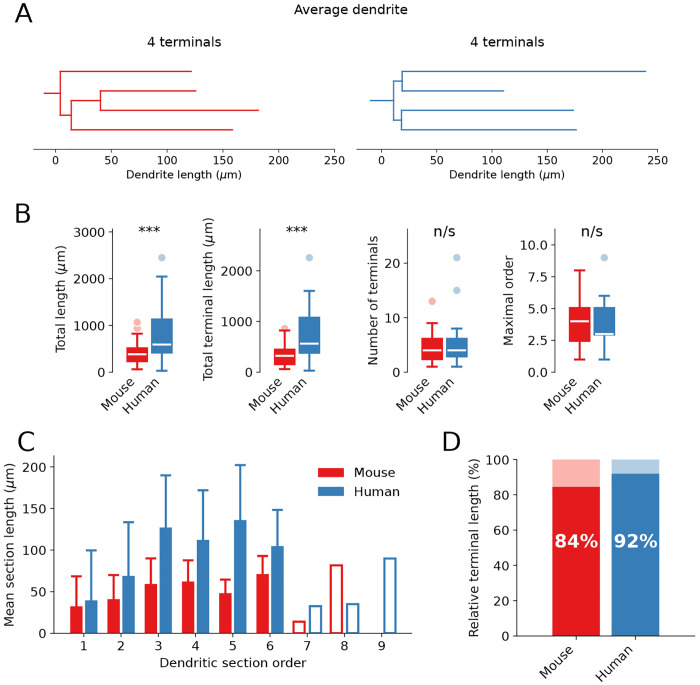
Morphometry of the dendritic branches. **(A)** Example dendrograms of the dendritic branches with average number of terminal segments (mouse in red, human in blue). **(B)** Boxplot diagrams for morphometric features of individual dendritic branches (red mouse, blue human, white line for median values, outliers shown with dots). Statistical significance p < 0.001 (***) and p > 0.05 (n/s). Total dendritic length and total terminal length are significantly longer in human SPNs (p = 0.00028 and p = 0.00008, respectively), while number of terminals and maximal order are similar (p = 0.74889 and p = 0.83002, respectively). **(C)** Mean length of the dendritic segment per topological order (order 1 for primary dendrites, etc.). Whisker lines for STD, unfilled boxes for single dendrites. **(D)** Relative length of the terminal segments normalised to the total length of the dendritic branch. The number of complete dendrites analyzed in (B-D) is 47 for mouse and 35 for human.

In the average SPN, a complete dendritic branch has four terminal segments in both mouse (red) and humans (blue), although the distal dendritic segments tend to be significantly longer in the human SPNs (Fig 3A). Each SPN neuron has on average 5 primary dendrites, whether mouse or human, and altogether around 30 terminal segments per complete dendritic arbour (Figs 3B, S4 and S5). Both the total length of the dendrites and the length of the terminal segments are significantly longer in human SPNs (p < 0.001), whereas the number of terminals and the maximal topological order are similar. There is, however, some variability. Thus, the SPN somata can emit from 3 to 9 dendrites (3–8 in mouse data), the median value is 6 in the human reconstructions and 5 in the mouse. Primary dendrites bifurcate to give rise to higher branch orders, the maximal order being 9 in human and 8 in mouse neurons in our datasets. The number of terminals per dendritic branch ( S4 and S5 Figs) varies between 1 (non-bifurcating primary dendrites), on average 4 and exceptionally (one case) up to 21 in human neurons (13 in mouse). The median value, however, is 4 terminals per dendritic branch in both species (mean values 4.4 and 4.6 in mouse and human, respectively).

Fig 3B and 3C provides the morphometry of the dendritic branches and shows that the number of terminals and maximal order of dendritic segments are similar, while the total dendritic length, the mean segment length and, particularly, that of the terminal segments is longer in human SPNs. The segment length for branch orders 1–6, starting from the segment closest to the soma (order 1, the primary dendrite), is shown in Fig 3C as mean and STD, and single instances for orders 7–9 are indicated by the open columns. Primary dendrites have similar length in both species, while higher order segments are longer on average in humans, particularly, due to the contribution of the terminal segments. The cumulative length of the terminal dendritic segment dominates entirely and represents 84% of the total dendritic length in mice and 92% in humans (Fig 3D), which means that the available dendritic area for synapses is essentially on the terminal dendritic segment.

**Fig 4:**
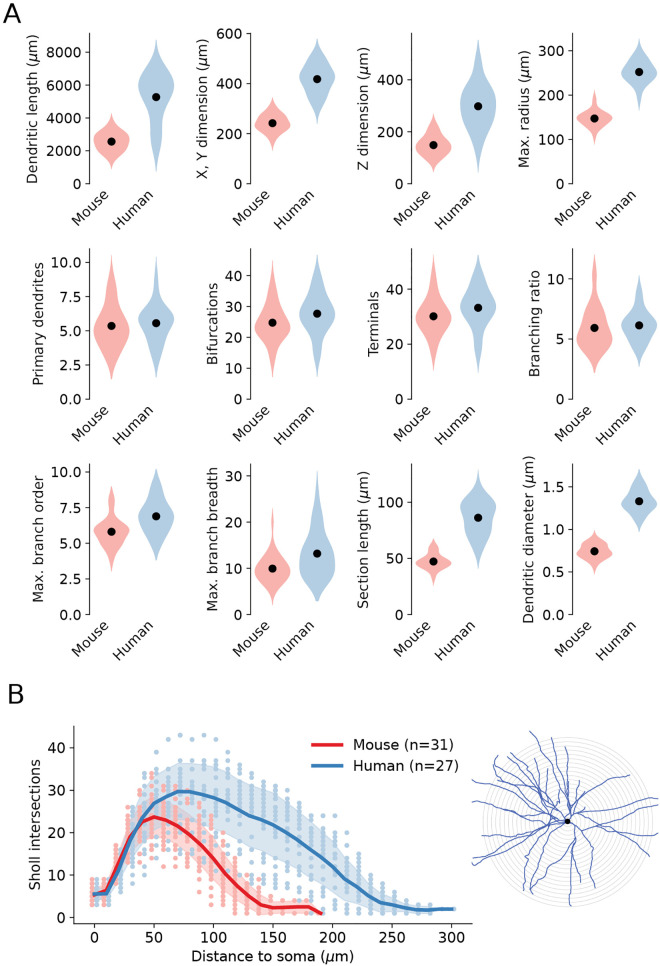
Morphometry of repaired SPN reconstructions. **(A)** Violin plot diagrams for neuronal morphometric features (dots for mean values). **(B)** Sholl diagram of SPN reconstructions. Solid lines for mean values, shaded areas for standard deviation and dots for individual reconstructions. Step of the Sholl radii is 10 μm (see inset). The number of reconstrcutions analyzed in (A-B) is 31 for mouse and 27 for human.

Repaired single cell morphological reconstructions retain all their intact dendritic branches as well as the original portions of the sliced dendrites expanded during the repair process. The overall morphometry of the repaired SPN reconstructions of human and mouse is detailed in Fig 4A, with the number of primary dendrites, bifurcations, terminals and branching ratios being similar (middle row), while the overall extension of the dendrites is larger in human SPNs (upper row). The number of primary dendrites shows a tendency to be higher in humans (median 6 versus 5 in mice) but this difference is not statistically significant. Maximal branch order and maximal branch breadth are more prominent in human cells. The Sholl analysis in Fig 4B shows a similar shape of human and mouse SPNs, but in humans the peak is higher, and it is more extended due to the longer terminals.

**Fig 5:**
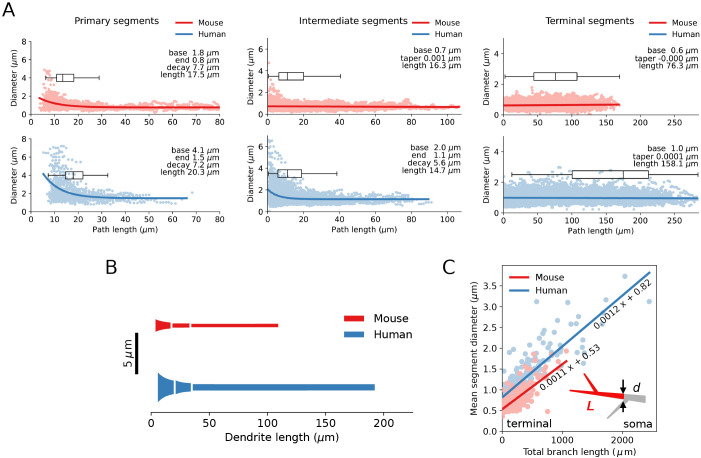
Dendritic diameter as function of distance to the soma. **(A)** Best fit for primary, intermediate and terminal dendritic segments, boxplots for segments lengths. Mean values are shown in the insets for base and end diameters, segment lengths, exponential decay or linear taper rates. Dots correspond to the reconstructed points of the complete dendrites (47 complete mouse dendrites and 35 complete human dendrites). **(B)** Mean diameter and mean length of the primary, intermediate and terminal segments of the complete dendrites (left to right, respectively). **(C)** Diameter of a given dendritic segment *d* is linearly proportional to the total dendritic length *L* of the distal part of a dendritic branch originating from that segment (see inset; arrows point out a dendritic node and corresponding dendritic subtree in red).

What about the diameter of the dendrites? The overall dendritic diameter is larger in human SPNs (bottom panels in Fig 5A). The terminal segments will have a major impact on the mean dendritic diameter as they represent 84% of the total dendritic length in mouse and 92% in human SPNs (Fig 3D). The terminal segments have the same diameter throughout their length, 0.6 μm in mice and 1.0 μm in humans. Non-bifurcating primary dendrites (i.e., which are also the terminal segments) are excluded from the analysis; likewise, intermediate dendrites do not include primary or terminal segments, respectively. The primary segments of the dendrites have a marked tapering, which also applies to the intermediate segments of humans, whereas the intermediate dendrites of mice have after initial tapering nearly the same dimension as the distal ones. Here, exponential and linear fit functions were used. Alternative approximations—such as a quadratic taper profile, which has been shown to provide optimal current distribution in branching passive cables [39]—are also possible. Dendritic diameter, in general, is a function of multiple factors such as the distance to soma, order of the dendritic segment, diameter of the parent segment etc., and it is known to vary with neuron type (e.g., reviewed by [40]). However, in SPNs it follows a linear dependence on the total length of the distal part of a dendritic branch originating in a given node, as shown in Fig 5C (also in [41–43]), similar to dendritic terminals of the cat motoneurons [44] or complete dendrites from the cat thalamocortical projection neurons [45]. This proportionality holds for both mouse and human dendrites. Fig 5B shows in a diagrammatic form an average mouse and human SPN dendrite with its primary, intermediate and terminal segments (only one segment after each bifurcation is shown).

### The distribution and shape of spines in human and mouse SPNs

**Fig 6:**
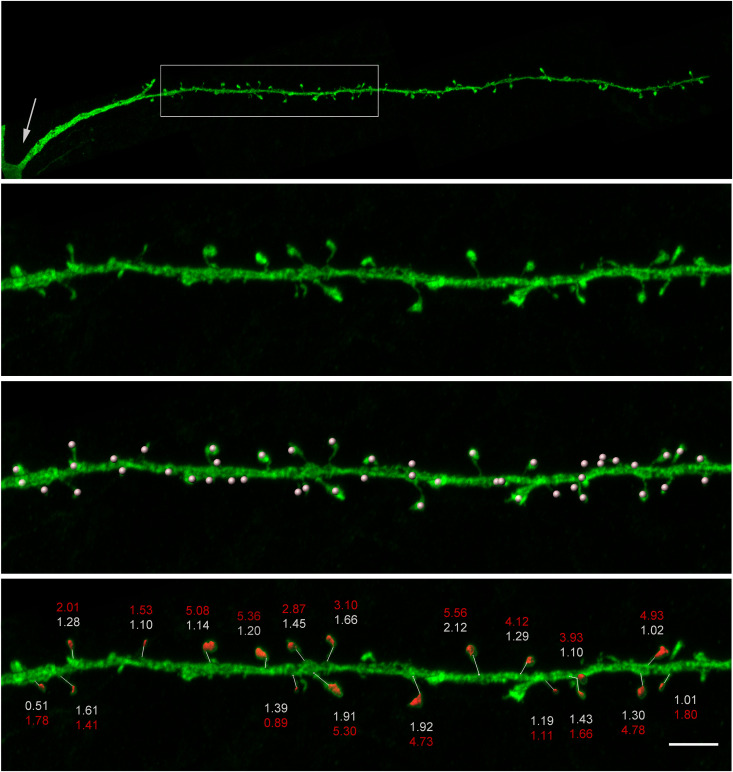
Reconstruction of the dendritic spines of human SPNs. **(A)** Confocal photomicrograph of a horizontally projecting dendrite from an intracellular injected human SPN. Soma is shown with an arrow, and the rectangle depicts the region of interest. **(B)** Magnified ROI. **(C)** Same image as in B to illustrate the position of spines. **(D)** 3D reconstructions of spines showing a clear head. Spine head area is shown in red, spine neck length in white numbers (see S1 Table for full statistics). Scale bar (in D) is 16 μm in A and 5 μm in B-D.

**Fig 7:**
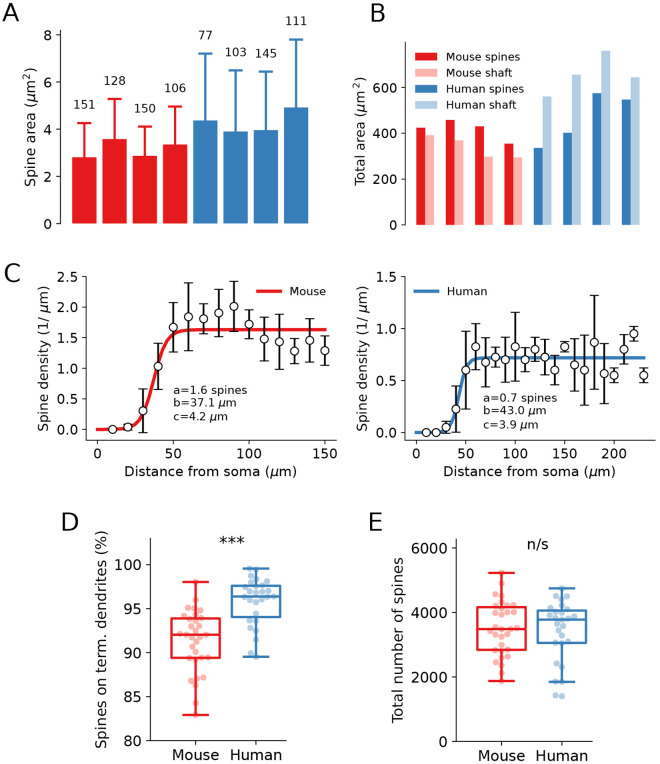
Characterisation of the dendritic spines. **(A)** Total spine area calculated in four reconstructed dendrites of mouse (red) and human (blue) neurons. Numbers correspond to the total number of spines per dendrite. **(B)** Total membrane area of dendritic spines and corresponding dendritic shafts in four reconstructed dendrites of mouse (red) and human (blue) neurons. **(C)** Dendritic spine density with distance from the soma. Parameters of the sigmoidal distribution (a, b, c) are given in the insets. **(D)** Proportion of spines located at the terminal dendritic sections (p = 0.000001). **(E)** Total number of spines per neuron (p = 0.67292) estimated for the repaired morphological reconstructions using spine density distributions from **(C)**.

A characteristic of the SPNs is the dense presence of spines (Fig 6) along the dendrites. Each spine has a head of varying size and a neck that can be of varying diameter and length that connects the spine head to the main dendritic trunk. Glutamatergic terminals mostly form one or few synapses on the spine head, but they can also be numerous along dendritic shafts [46,47]. In contrast, GABAergic terminals target mainly the dendritic shaft and are less commonly found on dendritic spines [47–49]. The average area of each spine is smaller in the mouse (3.1 μm^2^) than in the human SPNs (4.3 μm^2^; Fig 7A and S1 and S2 Tables). We investigated the correlation between spine size and distance from the soma using a non-parametric Spearman analysis, as the data were not normally distributed. The analysis revealed a weak but significant positive correlation in mice (r = 0.1273, p = 0.0032, n = 535). In contrast, no significant correlation was observed in humans (r = -0.03833, p = 0.4246, n = 436).

In addition, dendritic spine surface area was analyzed as a function of the distance from the soma (S6 Fig and S3 Table). The results showed that values were similar along the length of the dendrites in humans, whereas in mice there was a slight increase in spine surface area along the dendritic length.

In mice, the total membrane area of each dendrite is larger with regard to the spines as compared to the shaft (Fig 7B), while in humans the dendritic shaft dominates. Fig 7C shows that for the first 25 μm the spine density is near zero to increase to a maximal value around 50 μm from the soma and then remain largely at this level throughout the dendrite, sometimes with a tendency to decay with distance in both humans and mice. Spine distribution is modelled as a weighted sigmoidal function $S(x)=\frac{a}{\text{1}+e^{\frac{b-x}{c}}}$, where *x* is the path distance along the dendrite from a given point to the soma. Parameters (*a*, *b*, *c*) of the distribution are shown in the insets of Fig 7C for mouse and human dendrites, respectively (left and right panels). The spine density is higher in the mouse with around 1.6 spines/μm dendrite as compared to 0.7 spines/μm in human SPNs. The total membrane area occupied by spines (*A*_spines_) as compared to the dendritic shaft (*A*_shaft_) will thus differ between human and mouse dendrites. The total dendritic membrane area (*A*_total_) will be greater than *A*_shaft_ by a factor of *F*_spines_:


$$A_{\text{total}}=A_{\text{shaft}}+A_{\text{spines}}=A_{\text{shaft}}F_{\text{spines}}\;.$$

The *F*_spines_ factor is estimated in S7 Fig to be 2.23 and 1.70 for dendritic branches in mice and humans, respectively. This factor is important to consider during the simulations to account for the increased dendritic area due to the spines [50]. We incorporate the spine area by multiplying dendritic *C*_m_ by *F*(*x*) and dividing *R*_m_ by *F*(*x*), where *F*(*x*) is a function of path distance to soma $F(x)={\text{1}+(F}_{\text{spines}}-\text{1}fracS(x)a$. Parameter *a* in *F*(*x*) and *S*(*x*) was set to 1.0 and *F*_spines_ was set to 2.0 in our simulations of both mouse and human SPNs. The latter value agrees with that used by [51] (2.0) for pyramidal neurons in cortical microcircuits, [52] (2.0) for Purkinje cells and similarly by [50,53] (1.9) for L2/L3 pyramidal cells from human temporal cortex.

Distal location of the dendritic spines results in a distribution where most of the spines are placed on the terminal segments of the dendritic tree. We estimated the proportion of the spines on dendritic terminals using repaired SPN reconstructions and experimental spine density from Fig 7C and found that it exceeds 90% in both species (91.3% in mice and 95.8% in humans, as shown in Fig 7D). The total number of spines per reconstruction, however, was not significantly different (Fig 7E) which may be explained by the lower spine density that compensated for longer dendrites.

### Simulation of the somatodendritic arbor in mouse and human SPNs

Our previous models of mouse SPNs were based on a detailed knowledge of the somatodendritic morphology, membrane properties, ion channels expressed pharmacologically, and expression pattern based on single cell RNAseq. For human SPNs, we now have the detailed somatodendritic morphology with high resolution (Figs 1-7) and the demonstration that the human SPNs essentially appear as enlarged versions of the mouse SPNs. In addition, we know the ion channel gene expression patterns in humans [31], as shown in S8 Fig. There is a very similar pattern to that of mouse SPNs [30]. Available extracellular recordings from non-human primates and human patients confirm a very low baseline activity (< 2Hz) in the primate striatum as in rodents [21,22,54,55]. Single-cell intracellular electrophysiological data from human striatal neurons are not available. We therefore can only model the human SPNs, based on their morphology and assuming a similar expression pattern of ion channels and the level of excitability as in the mouse (see Discussion). We have used the same strategy as for the mouse with multi-compartment representation of electrically active plasma membrane populated with ion channels of Hodgkin-Huxley type as in Hjorth et al [33]. Dendritic spines are not directly included, but the membrane area of the spines is accounted for (see above). The density of ion channel conductances (S9 Fig) in human SPN was estimated as in the previously developed mouse model [41,33] and extended by 70% to account for the increased dendritic dimensions.

**Fig 8:**
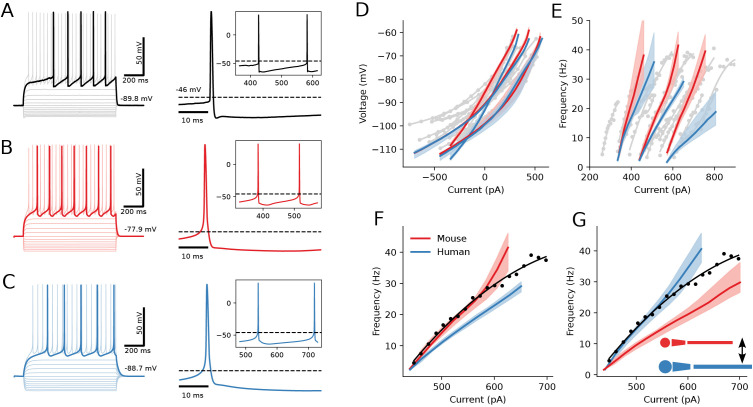
Simulation of the detailed biophysical models of SPNs for mouse (red) and human (blue). **(A)** Experimental current clamp recording from the mouse D1-positive SPNs. **(B-C)** Simulated neuron models optimised to the experimental data (A) using mouse (B) and human (C) morphologies. **(D-E)** Ensembles of validated models optimised to three selected SPNs, experimental data plotted in grey. Subthreshold current-voltage responses (D) and suprathreshold discharge rates (E) are shown. Shaded areas display the range of the values within each ensemble. **(F)** Magnified current-frequency relation for one set of models (from the middle of **(E)**). Black color for experimental data, red for the mouse and blue for the human morphologies. **(G)** Current-frequency relation with exchanged morphologies, mouse SPN reconstruction is scaled to human proportions (retains red color) and human reconstruction is downscaled to mouse dimensions (retains blue color). Rescaled mouse and human SPN reconstructions behave as valid replacements of each other in electrophysiological simulation.

Fig 8A shows an experimental recording from a mouse SPN and the corresponding simulated models of mouse and human SPNs are shown in Fig 8B and 8C. The spike pattern during intracellular stimulation and the details of the action potential and the interval to the next action potential are very close to that of the recorded neuron in Fig 8A. The resulting models faithfully reproduce critical electrophysiological features of SPNs, including also the resting membrane potential at a relatively negative level, strong inward rectification, first-spike latency, moderate after-hyperpolarisation and stable action potential amplitude (Fig 8A-C).

Mouse neuron models were optimised for the best match of the experimental features. Human neuron models were mainly optimised for the same excitability, i.e., the rheobase current and the firing rate close to the rheobase, together with major action potential features. Fig 8D and 8E shows the subthreshold and suprathreshold responses to different positive or negative current stimuli in biological data (grey) and model neurons of mouse (red) and human (blue). In all optimisation cases the best human SPN models consistently exhibited lower discharge rates than their mouse counterparts for somatic current injection, as shown in Fig 8E and 8F. Slower firing responses combined with the same excitability were previously reported for human layer 2/3 cortical pyramidal neurons by Beaulieu-Laroche et al [56] and Purkinje cells by Masoli et al [52].

An important morphological feature of SPNs is that the number of primary dendrites is similar in humans and mice. This contrasts with human pyramidal and Purkinje cells, where the number of primary dendrites is greater than in the same types of neurons in mice [57,52]. This similarity makes SPNs more comparable between humans and mice, suggesting the potential to more easily reuse mouse and human SPN reconstructions in simulations interchangeably, with appropriate spatial scaling (Fig 8F and 8G), to explore how this affects cellular properties. To verify this, we rescaled the mouse reconstruction to human dimensions and downscaled the human morphology to mouse proportions and then rerun the optimisations for both models. We uniformly increased dendritic diameters of the mouse SPN by a factor of 1.7 and doubled the length of the terminal segments. Correspondingly, the human SPN reconstruction became thinner in dendrites by 0.6 and the terminal segments were shrunk by 50% in length. Spherical somata were swapped between the two reconstructions. The resulting morpho-electric models, re-optimized against the original electrophysiological data, display the behaviour of the opposite species as shown in Fig 8G. Mouse SPN morphology brought up to human size shows slower firing rates as if they were true human reconstructions and, conversely, human reconstructions reduced to mouse dimensions demonstrate faster spiking.

**Fig 9:**
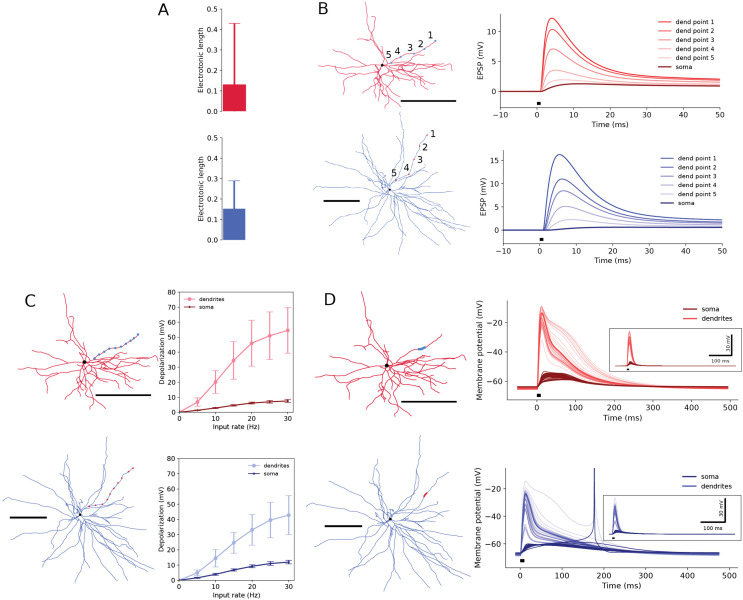
Electric properties of the dendritic terminal segments of the mouse (red, 35 terminals) and human (blue, 37 terminals) neuron morphologies in corresponding computational neuron models. **(A)** Electrotonic length of the terminal segments. **(B)** Shape of EPSP in the soma (dark line) and in the terminal dendritic segment (light lines) at different locations on the terminal dendritic segment (points 1-5). The glutamatergic synapse is located at the tip (point 1). **(C-D)** Response to simulated glutamatergic synaptic inputs, AMPA and NMDA. **(C)** Distributed asynchronous synaptic drive. Left: 10 synapses placed uniformly along the terminal segment; right: membrane depolarisation in the dendritic segment (light lines) and soma (dark lines), error bars for STD, variability for all terminals. **(D)** Clustered synchronous synaptic drive. Left: synapses are placed in the middle of a terminal segment and stimulated in sequence with 1 ms interval; right: EPSPs in the dendritic segment (light lines) and soma (dark lines). Prolonged NMDA-spike in a dendrite may trigger action potential in the soma (truncated). Inset, shows the effect of setting the NMDA part to 0. Scale bars in (B-D) are 100 μm.

Backpropagation of the electrical signals throughout the dendritic branches, from the soma towards the periphery, is to a large degree determined by the effective electrotonic length of the dendrites, particularly their terminal segments which are dominating in the SPNs. The electrotonic length of the terminal dendrites $L=\frac{l}{\lambda }$, where *l* is the physical length and $\lambda =\sqrt{\frac{R_{\text{m}}d}{\text{4}R_{\text{a}}}}$ is the space constant for diameter *d*, appears to be similar for both human and mouse computational neuron models despite the difference in extension (Fig 9A). This is explained by the larger dendritic diameter *d* and the specific membrane resistivity *R*_m_ as suggested by the model optimisation, assuming specific axial resistivity *R*_**a**_ constant. As a result, the decay time constant for the local membrane potential perturbations in the terminal segments of dendrites appears to be greater too, 10.47 ± 1.94 ms in n = 37 dendrites against 6.81 ± 0.16 ms, n = 35, in mice calculated at the resting membrane potential. In Fig 9B, the signal propagation in the opposite direction, i.e., from dendrite to soma, is simulated. We stimulate a glutamatergic synapse (AMPA and NMDA) at the tip of a dendrite and calculate the size of the EPSP as it is transmitted along the terminal dendrite to the soma. Synaptic conductance in the human model is increased by 37% in proportion to the larger spine area (4.263/3.118). As expected, there is a marked reduction of the amplitude of the EPSP, and the degree of attenuation is very similar in human and mouse SPNs. Fig 9C shows another condition when EPSPs are applied asynchronously at different rates (5–30 Hz) to multiple locations distributed uniformly along the terminal dendrite. The average depolarisation is reported in the middle position of the dendrite and in the soma. The level of depolarisation in the dendrite and soma is smaller in the human neuron, but in general comparable as a result of the equivalent temporal and spatial summation in both models. This means that the human and the mouse SPNs are “designed” to behave in a similar way to synaptic input despite the difference in dimensions. Fig 9D illustrates another condition, with synchronously activated clustered synapses placed in the middle of the terminal segment, which will amplify the response locally and resemble the situation that may occur with excitatory input that will target a certain location on the dendrite. Plateau potentials are a ubiquitous occurrence in the brain [58] and have also been demonstrated in the striatum [59,60]. Sequential activation of 10 synapses with a 1 ms interval, leads to a marked local depolarisation initially at around 50 mV and lasting over 200 ms. The depolarisation gradually declines and is due mainly to the voltage-dependent NMDA receptors that remain open and only gradually will close. The inset shows that if NMDA receptors are blocked the duration of the response is markedly reduced. At the soma level the depolarisation is smaller (around 10 mV) and lasts as long. It can trigger an action potential. Again, the response pattern is similar in human and mouse SPNs.

In conclusion, the simulations suggest that the human SPNs will respond in a similar way to the mouse SPNs, and therefore the mouse SPNs can be considered a valid model applicable to the human SPNs.

## Discussion

### The somatodendritic structure of SPNs

The purpose of this study was to describe the somatodendritic characteristics of human SPNs in some detail to allow for a comparison with the SPNs of the mouse. Overall, despite the notably larger size of human neurons, the somatodendritic tree of human SPNs is relatively similar in basic design to that of mouse SPNs, particularly in terms of the number of primary dendrites and the general structure of the dendritic arbour. However, Sholl analysis of repaired reconstructions indicate that the complexity of the dendritic arbor may be somewhat higher in humans. Furthermore, we found that the dendrites have the same average number of terminal segments (four), although the length of the terminal segments is longer in humans. The latter occupies 84% of the length in mice and 92% in humans, which is where most of the synaptic input will be located. Moreover, the diameter of these terminal segments is thin and remains the same throughout the terminal in both species, 1.0 μm in humans and 0.6 μm in mice. In contrast to SPNs, findings in the cerebral cortex and cerebellum, where human pyramidal neurons and Purkinje cells exhibit greater complexity and cannot simply be considered scaled-up versions of their mouse counterparts. These human neurons demonstrate a higher degree of voltage compartmentalisation and enhanced computational capacity [43,50,52,53,56,61]. Nevertheless, these studies also report functional behaviours comparable to those of rodent neurons, particularly regarding the rheobase current and action potential shape. In summary, although this study did not reveal the major differences in somatodendritic morphology previously described in other brain regions, this outcome may reflect the broad scope of our analyses and the relatively limited number of neurons examined, which may have restricted our ability to detect more subtle differences. Nevertheless, these findings provide a valuable baseline and highlight the importance of future studies with higher resolution and greater sampling depth.

The distal terminals are studded with dendritic spines throughout, while the primary dendritic segment close to the soma are devoid of spines for the first 25 μm and the density of spines is at its maximal level at 50 μm and beyond, again similar in the two species. The average spine size is larger in humans than in mice (4.3 versus 3.1 μm^2^). Moreover, the expression of ion channels in human SPNs, as evaluated by single cell RNAseq, is similar (S8 Fig; [30,31]) to mouse SPNs. These findings taken together, suggest that it should be possible to get a handle on the cellular properties of human SPNs through modelling them based on the detailed description we have provided in the mouse SPNs and that we modelled in great detail previously [33]. Future studies incorporating age-matched comparisons across species would be valuable to more accurately assess these differences.

### In silico analysis of human SPNs

In the simulations we used the morphology of mouse and human SPNs with detailed dimensions of dendrites. Moreover, from single cell RNAseq we know that the expression of different ion channels is similar in the two species. It is therefore reasonable to populate the model SPNs of humans and mice with a similar set of ion channels. We could then see that they respond in a similar way (Fig 8), but that in response to current injection the human cells consistently were somewhat less responsive. This can be explained simply by the dimensions of the somatodendritic tree, since if the mouse morphology was scaled up to human SPN dimension it responded as a human SPN, everything else being unchanged. Conversely, if the human SPNs were reduced to mouse dimensions they were more responsive and behaved as a mouse SPN (Fig 8G). This data, on one hand, supports the notion that the human SPN is essentially an enlarged counterpart of the mouse SPN and suggests that the difference in dimensions leads to a consistent change in responsiveness to current injection. Scaling is not linear or consistent across all aspects of the neuron, however. Various neuronal components scale according to distinct rules, shaped by the demands of larger and more complex brains.

With simulation one can perform analyses that are difficult to carry out experimentally and show how an AMPA/NMDA synapse located at a distal site is dramatically attenuated and delayed on its way to the soma, again with a similar response pattern in the two types of SPNs. However, the effect is somewhat more pronounced in the human SPN again explained by the difference in dimensions. Interestingly, clustered synapses (NMDA/AMPA) can give rise to a large depolarisation followed by a slowly decreasing depolarisation, which is entirely dependent on the NMDA channels. Such plateau-like potentials can be important in the context of synaptic plasticity, and in any case, they provide a substantial depolarisation of the soma.

That human SPNs are larger in dendritic dimensions than mouse SPNs implies that they will be able to receive input from a more extensive area of projecting fibres. In this context it may also be pertinent to note that the volume of the human striatum is roughly 3 orders of magnitude larger than that of the mouse (35000 mm^3^ versus 23 mm^3^), which of course places other demands on the organisation of the connectivity.

In conclusion, our data demonstrate that similar basic design principles of SPNs apply to both humans and rodents. These findings further support the use of mice as a model system for studying many aspects of the human SPNs, as well as the neurological and psychiatric disorders that affect the basal ganglia.

## Materials and methods

### Ethical statement

Human brain tissue was obtained at autopsy from the Unidad Asociada Neuromax—Laboratorio de Neuroanatomía Humana, Facultad de Medicina, Universidad de Castilla-La Mancha, Albacete, Spain, and the Laboratorio Cajal de Circuitos Corticales, Universidad Politécnica de Madrid–Consejo Superior de Investigaciones Científicas (CSIC), Madrid, Spain. The tissue was obtained in compliance with national laws and international ethical and technical guidelines on the use of human samples for biomedical research purposes.

All experimental protocols involving the use of animals were performed in accordance with the Ethical Committee for Research of the Spanish National Research Council (CSIC) and the Autonomous University of Madrid following institutional, Spanish and European guidelines (Boletín Oficial del Estado (BOE) of 18 March 1988; and 86/609/EEC, 2003/65/EC European Council Directives).

### Tissue preparation

#### Human brain samples.

Samples were obtained at autopsy (4 h post-mortem) from the Unidad Asociada Neuromax — Laboratorio de Neuroanatomía Humana, Facultad de Medicina, Universidad de Castilla-La Mancha, Albacete; Laboratorio Cajal de Circuitos Corticales UPM-CSIC, Madrid, Spain. The tissue was obtained following national laws and international ethical and technical guidelines on the use of human samples for biomedical research purposes. In the present study, we used 2 human cases (age, sex): AB5 (59, M), AB6 (92, F) with no evidence of cognitive impairment or dementia. These cases were used as controls in a previous study unrelated to the present investigation [62]. Upon removal, the brains were immersed in cold 4% paraformaldehyde in 0.1 M phosphate buffer, pH 7.4 (PB) and sectioned into 1.5-cm-thick coronal slices. Small blocks from the caudate and putamen regions (Fig 1A and 1B; Allen Human Brain Reference Atlas; https://atlas.brain-map.org/) were then transferred to a second solution of 4% paraformaldehyde in PB for 24 h at 4°C. Vibratome sections (300 μm thick) were obtained in the coronal plane, (x, y, z) axes corresponding to left-right, ventral-dorsal and posterior-anterior orientations, respectively.

#### Mouse brain samples.

Two male 8-week-old and four male 6 months-old C57Bl/6 mice were used in this study. They were housed at room temperature under a 12 h light-dark cycle. Food and water were provided *ad libitum*. FluoroGold retrograde injections (see below) were performed in the male 8-week-old C57Bl/6 mice.

The mice were deeply anaesthetised with an intraperitoneal injection of pentobarbital (40 mg/kg) and intracardially perfused with 200 mL of freshly prepared fixation solution (4% PFA in PB). The brains were postfixed for 6–16 h (overnight) in the same fixation solution. Vibratome sections (150 μm thick) were obtained in the coronal plane, same orientation as in human samples. Section thickness was adapted to neuronal size in each species and optimized to achieve high-quality confocal imaging.

### FluoroGold retrograde injections

This experiment was performed in the male 8-week-old C57Bl/6 mice. Striatonigral neurons were retrogradely labelled by unilateral injections of FluoroGold (sc-358883, Santa Cruz Biotechnology, Inc. Dallas, Texas, USA) into the substantia nigra (anteriority from bregma (mm): ₋ 3.3; laterality: ₋ 1.3, depth from cortical surface: −4.5) (S1 Fig). Animals were intraperitoneally anaesthetized with 8 mg/kg ketamine and 1.2 mg/kg xylazine, and 0.5 μl of FluoroGold (2% dissolved in 0.9% weight/vol NaCl) was injected unilaterally using a glass micropipette (tip diameter 30 µm). Overlying skin was sutured, atipamezole hydrochloride was injected subcutaneously (20mg/Kg) to reverse sedative effects, and mice were returned to their home cages after recovery from anaesthesia. Seven days after injection, animals were deeply anaesthetized with an intraperitoneal injection of pentobarbital (40 mg/kg) and intracardially perfused with 200 mL of freshly prepared fixation solution (4% paraformaldehyde in phosphate buffer (PB)). The brains were postfixed for 6–16 h (overnight) in the same fixation solution. Vibratome coronal sections (150 μm thick) were cut.

### Intracellular injections and immunocytochemistry

Sections were prelabelled with 4,6-diamidino-2-phenylindole (DAPI; Sigma, St Louis, MO), and a continuous current was used to inject individual cells with Lucifer yellow (8% in 0.1; Tris buffer, pH 7.4; LY) in the caudate and putamen regions of the human brain and in the striatum region of the mouse (Fig 1). In the mice striatum, LY was intracellularly injected into selected D1 SPNs, as identified by Fluorogold labelling (S1 Fig). LY was applied to each injected cell by continuous current until the distal tips of each cell fluoresced brightly, indicating that the dendrites were completely filled and ensuring that the fluorescence did not diminish. Following the intracellular injections, the sections were immunostained for LY using rabbit antisera against LY (1:400 000; generated at the Cajal Institute) diluted in stock solution (2% bovine serum albumin, 1% Triton X-100, and 5% sucrose in PB). The sections were then incubated in biotinylated donkey anti-rabbit IgG (1:100; Amersham, Buckinghamshire, United Kingdom) and streptavidin-conjugated Alexa fluor 488 (1:1000; Molecular Probes, Eugene, OR, U.S.A.). Finally, the sections were washed and mounted either with ProLong GoldAntifade Reagent (Invitrogen Corporation, Carlsbad, CA, USA) or with 50% glycerol in PB. See Elston et al. (2001) and Benavides-Piccione et al. (2020) [63,64] for further details of the cell injection methodology.

### Cell reconstruction and quantitative analysis

Sections were imaged with confocal microscopy with a ZEN inverted scanning confocal system (Zeiss LSM 710; Carl Zeiss Microscopy GmbH, Jena, Germany) using a 488 nm Argon laser and 405 nm UV and the fluorescence of DAPI and Alexa 488 were recorded through separate channels. Consecutive stacks of images (×40 oil, voxel size, 0.231 × 0.231 × 0.29 μm^3^ for the human and ×63 oil, voxel size, 0.082 × 0.082 × 0.28 μm^3^ for the mouse) were acquired to capture the whole dendritic arbor of the cell. To analyze dendritic spines, dendrites were imaged using consecutive image stacks (×63 oil, voxel size, 0.073 × 0.073 × 0.28 μm3) spanning from the soma to the distal tip of the dendrite. Since intracellular injections of the SPNs were performed in coronal sections (150–300 μm-thick), the part of the dendritic arbour nearest the surface of the slice from which the cell soma was injected (typically at a depth of ∼30–50 μm from the surface) was lost. Using a similar method of intracellular injection, Krimer et al. [65] estimated that the reconstruction of neurons represented approximately two–thirds of the total dendritic arbour of injected cells.

Data points of neuron morphology of each SPN included in the analysis (27 SPNs from 2 human individuals and 31 SPNs from 6 mice) were extracted in 3D using Neurolucida 360 (MicroBrightfield). Briefly, the dendrites were described through 3D points, delimiting the different segments that form the cell arbour. These points have an associated diameter that provides the information of the varying thickness of the dendrite along its length. Dendritic morphological variables were extracted using Neurolucida software as total values, per branch order and per distance from the soma. In a few cases an axon initial segment was reconstructed (n = 14, length of reconstructed axon 23–759 μm).

Dendritic spine density was also analysed. Spines were counted on 10 horizontally projecting dendrites, randomly taken from different cells of the 6-month-old mice. Spine density was calculated every 10 μm from the soma to the distal tip of the dendrites using Imaris 6.4.0 (Bitplane AG, Zurich, Switzerland). Spine structure was analysed using the same software. To capture dendritic spine and shaft surface area, a specific threshold was selected to constitute a solid surface that exactly matched the contour of each dendritic spine and shaft. However, occasionally multiple intensity thresholds were needed to capture the complete morphology of a dendritic spine, which could potentially affect measurement accuracy. To address this, Neuronize v2 [66] was used to convert the separate sub-meshes into a single, corrected, unified mesh, from which spine surface area was then calculated. In addition, we measured the spine neck length, spine neck diameter and spine head surface area in a selection of 150 spines per group that showed a clear head (Fig 6). The spine neck length and spine neck diameter were manually marked in each selected dendritic spine from the point of insertion in the dendritic shaft to the spine head, while rotating the image in 3D.

### Computational modeling

#### Digital processing of morphological reconstructions.

Morphological reconstructions were converted from Neurolucida ASC file format to SWC using the HBP Neuron Morphology Viewer (https://neuroinformatics.nl/HBP/morphology-viewer-dev) and adjusted to comply with standardised SWC format [67]. Somata contours were replaced with spheres using mean values for the centre and radius. Adapted reconstructions were centred at origin and corrected for tissue shrinkage and z-jumps, if necessary [36]. Shrinkage correction factor was 1.7 in *z*-axis for sections mounted in ProLong, sections in 50% glycerol did not need correction, also no correction was applied in (*x, y*)-plane.

To deal with the slicing artefacts, the cut points of the dendrites were located in the vicinity of the upper surface of the slice (20 μm and 10 μm for mouse and human reconstructions, respectively) and a pool of intact dendritic branches not containing the cut points was collected (termed ‘sanitized’ in data repository). Additionally, a set of half-reconstructions digitally sliced at root in (x, y)-plane was created to generate predictions for repair. During repair of a reconstruction, for each dendritic cut point, an intact dendritic branch of the same topological order was randomly selected from the intact pool, then copied and attached to the damaged dendrite. Continuity of the dendritic diameter over the repaired connection was ensured by scaling the diameters of the repair branch (from its base to the tips) to the mean diameter of the cut segment. segments that become too thin or too thick after the scaling, were discarded (the minimal and maximal diameters are taken from the terminal segments of the intact reconstructions, [0.376; 0.876] microns for mouse and [0.59; 1.26] microns for human). This is repeated N times for each reconstruction (here N = 100). Assuming spherical symmetry of the reconstructed SPNs on average, we predict morphometry of the repaired neurons using their half-reconstructions and select the best matching repaired candidate.

Repaired reconstructions were resampled with fixed spatial resolution of 3 μm and upscaled by 1% to recover the dendritic length lost due to the resampling. Remaining digital artefacts (e.g., too short dendritic segments or misplaced nodes) were corrected manually for the final release.

All morphology manipulations as well as the morphometric measurements were done with the Python module treem (https://github.com/a1eko/treem) combined with Bash Shell and Python v.3 scripts.

#### Statistical analyses.

Statistical analyses were performed using Welch’s t-test from the statistical functions of SciPy v.1.10 stats module. Values p < 0.001 (***), p < 0.01 (**) and p < 0.05 (*) were considered statistically significant, exact p-values being given in the text. No data were excluded from the analyses. Boxplot visualisation uses Matplotlib v.3.6 implementation with medians, box extending from the first quartile (Q1) to the third quartile (Q3) of the data, whiskers extending from the box to the farthest data point lying within 1.5x the interquartile range (IQR) from the box and flier points past the end of the whiskers.

#### Biophysical neuron models.

Single-cell computational models were built using detailed three-dimensional reconstructions of neuron morphology corrected for slicing artefacts, experimental electrophysiological patch-clamp recordings and mathematical models of the ion channels of Hodgkin-Huxley type. Selection and distribution of ion channels were taken from the previous publications [41,33]) and re-optimized for newly acquired morphologies. The Blue Brain Python Optimization framework (BluePyOpt) by Van Geit et al., 2016, [68] was used for setting the free model parameters as described in Hjorth et al., 2020 [33] (see also Supplementary Information). Fixed values for temperature 35°C, base value of the specific membrane capacitance before correction for the total spine area 1 μF/cm^2^ (and up to 2 μF/cm^2^ following a sigmoidal distribution for dendrites after correction), cytoplasmic resistivity *R*_a_ = 150 Ohm·cm, reversal potential for sodium +53.34 mV and potassium -105.9 mV were applied globally. Leak conductance and reversal potential were constrained by a series of protocols with subthreshold somatic current injections and then fixed for optimization of the current-discharge properties, individually for each neuron model. Best candidate models (up to 10) returned by the optimizer are validated against the features extracted from the experimental recordings of the target neuron population (n = 13): RMP, input resistance, base firing rate, frequency-current slope, AHP depth, AP peak, amplitude and threshold. Also changes of the dendritic calcium concentration evoked by the back-propagating AP were checked, as in [41] and [33]. The model is considered valid if all measured features fall within 3 SD of the population mean.

## Supporting information

S1 Fig (A) Low power photomicrograph of a fluorogold injection in the mouse substantia nigra pars reticulata. (B-E) Four images taken from the same microscopic field of the ipsilateral striatum from the same animal as in A. (B) The section was stained with DAPI and retrogradely labelled Fluorgold SPNs indicated by arrows. Arrowheads point at DAPI-stained neurons not retrogradely labelled by Fluorogold. (C) The section was transferred to the stage of an inverted fluorescence microscope to intracellularly inject Lucifer yellow into selected SPNs, as identified by Fluorogold labelling (arrows). (D) Lucifer yellow injected D1 SPN. (E) Low magnification photomicrograph showing various Lucifer yellow D1 SPNs. Scale bar, shown in D, indicates 1250 µm in A, 15 µm in B-D and 400 µm in E.
(TIF)

S2 Fig Nomenclature of dendritic morphometry. *Dendritic arbor* - the whole set of dendrites of the neuron. *Dendritic branch* - the subset of dendrites that extends directly from the soma. *Dendritic segment* - a portion of a dendrite between two structural points (e.g., two branching points, soma and a branching point or a tip). *Primary dendrite* - initial, main dendritic segment that originates at the soma. *Terminal segments* - the most distal segments of a dendritic branch, not undergoing further branching. *Branch order* - hierarchical level of a dendritic segment within the tree in centrifugal order; primary dendrite is assigned branch order 1, secondary segments have branch order 2 and so on, with higher numbers indicating more distal segments from the soma. *Branch breadth* - the total number of terminals of a given branch, defines the hierarchical level of a segment in centripetal order; the terminal segments have breadth 1, the next segments after the branching points in the direction towards the soma have breadth 2, and so on; breadth of a given segment equals the sum of the breadths of its daughter branches.
(TIF)

S3 Fig Validation of the repair process for mouse SPNs. Distributions of morphometric features of the repaired reconstructions (dark red, 165 dendrites in 31 reconstructions) is compared to morphometry of the complete dendrites (light red, 47 complete dendrites). Z-scores are shown in bold for each morphometric feature.
(TIF)

S4 Fig Dendrograms of the dendritic branches of different topological breadth (number of terminals). Red for mouse, blue for human, the branch breadth number is stated to the left of each dendrogram. Only one example dendrite is shown for each breadth. In the current dataset, the mouse dendrites have breadth 1 (n = 8), 2 (n = 4), 3 (n = 6), 4 (n = 9), 5 (n = 6), 6 (n = 4), 7 (n = 4), 8 (n = 4), and 13 (n = 1). Human dendrites have breadth 1 (n = 4), 2 (n = 4), 3 (n = 9), 4 (n = 6), 5 (n = 2), 6 (n = 5), 7 (n = 1), 8 (n = 2), 15 (n = 1), and 21 (n = 1).
(TIF)

S5 Fig Dendrograms of the average repaired single-cell reconstructions. The repaired reconstructions of human SPNs are similar in shape and structure to those of the mouse with 5–6 primary dendrites (mean values 5.4 and 5.6 for mouse and human, respectively) and about 30 terminals per neuron on average (30.1 and 33.2).
(TIF)

S6 Fig Surface area (μm^2^) of the dendritic spines as a function of distance from soma along the dendrite (mean and SEM) for the human and mouse neurons (see also S3 Table).
(TIF)

S7 FigFactor $F_{\text{spines}}=\frac{A_{\text{spines}}+A_{\text{shaft}}}{A_{\text{shaft}}}$ estimated for four selected dendrites in mouse (2.23 ± 0.15) and human (1.71 ± 0.12) neurons.(TIF)

S8 Fig Comparison of the ion channel gene expression patterns in mouse and human SPNs (red and blue palettes, respectively). Data from Saunders et al. [30] for mice and Siletti et al. [31] for humans.
(TIF)

S9 Fig Distribution of electric conductances along the dendrites with distance from soma as in Hjorth et al. [33]. Here pas stands for the passive membrane conductance and membrane capacitance *C*_m_, naf for the fast Nav ion channel, kaf and kas for the fast and slow components of A-type K^+^ channel, and cat for a T-type Ca^2+^ ion channel. Conductances of the active ion channels in the human model are extended by 70%. Distance dependence of passive conductance and membrane capacitance is fitted to the spine density distribution in mouse and human reconstructions. The *F*_spines_ factor to account for the membrane area of spines is set to 2.0 in both models.
(TIF)

S1 Table Spine dimensions of the human striatal projection neurons (see Fig 6 for illustration).
(TIF)

S2 Table Surface area (μm^2^) of the dendritic spines and the corresponding dendritic shaft in human and mouse dendrites, 4 dendrites each (see Fig 7).
(TIF)

S3 Table Numerical values for S6 Fig on the dependence of the spine surface area (μm^2^) on the distance from soma (μm); mean and standard deviation, *N* is the number of spines reconstructed at a given distance from the soma.
(TIF)
