# Supplementary figures and images for: Mouse and human striatal projection neurons compared - somatodendritic arbor, spines and in silico analyses
Source: PLoS Comput Biol. 2025 Oct 9;21(10):e1013569. doi: 10.1371/journal.pcbi.1013569 (PMC12530558; doi:10.1371/journal.pcbi.1013569)

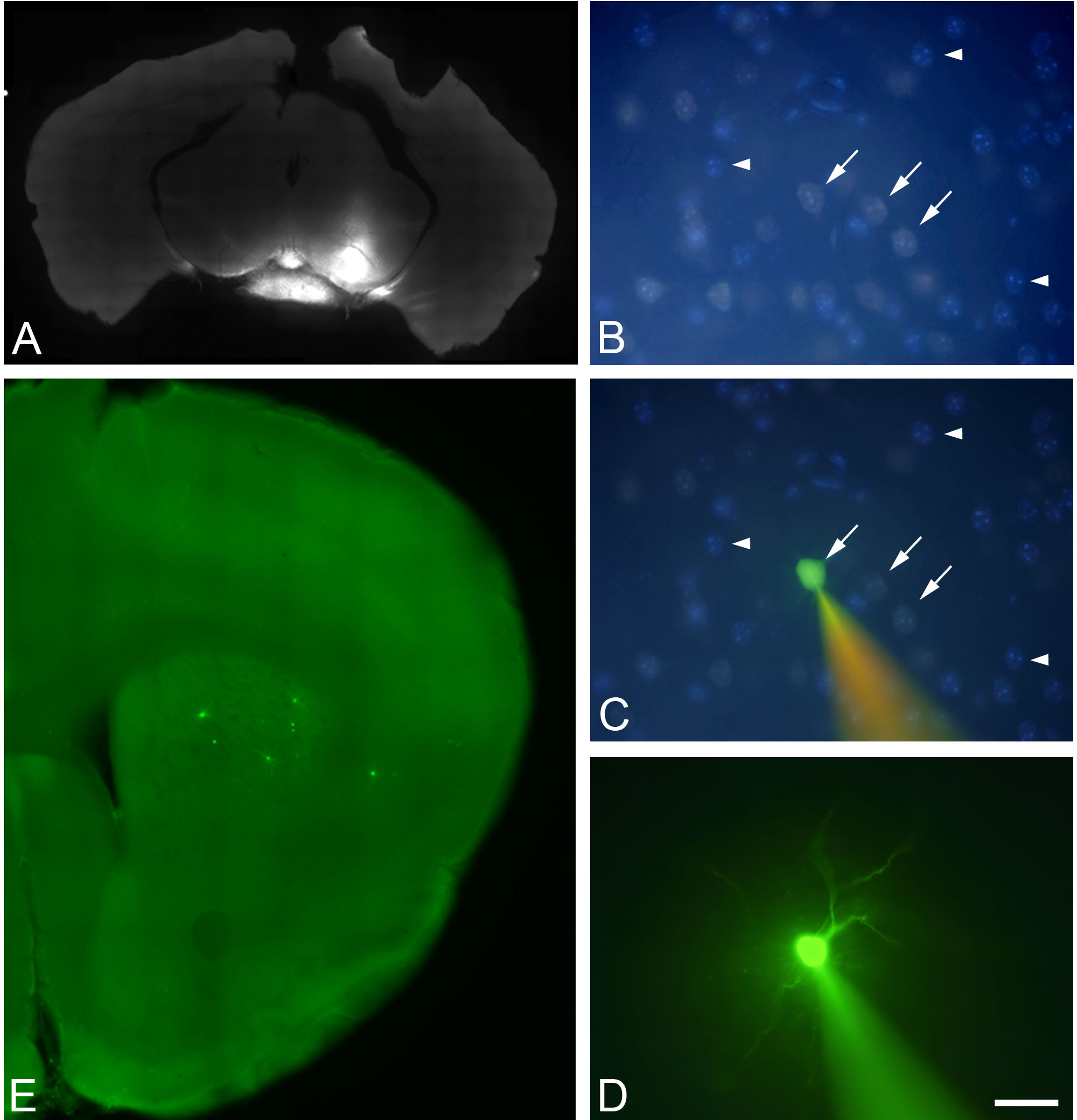

Supplement: S1 Fig — (A) Low power photomicrograph of a fluorogold injection in the mouse substantia nigra pars reticulata. (B-E) Four images taken from the same microscopic field of the ipsilateral striatum from the same animal as in A. (B) The section was stained with DAPI and retrogradely labelled Fluorgold SPNs indicated by arrows. Arrowheads point at DAPI-stained neurons not retrogradely labelled by Fluorogold. (C) The section was transferred to the stage of an inverted fluorescence microscope to intracellularly inject Lucifer yellow into selected SPNs, as identified by Fluorogold labelling (arrows). (D) Lucifer yellow injected D1 SPN. (E) Low magnification photomicrograph showing various Lucifer yellow D1 SPNs. Scale bar, shown in D, indicates 1250 µm in A, 15 µm in B-D and 400 µm in E. (TIF) [file pcbi.1013569.s001.tif]

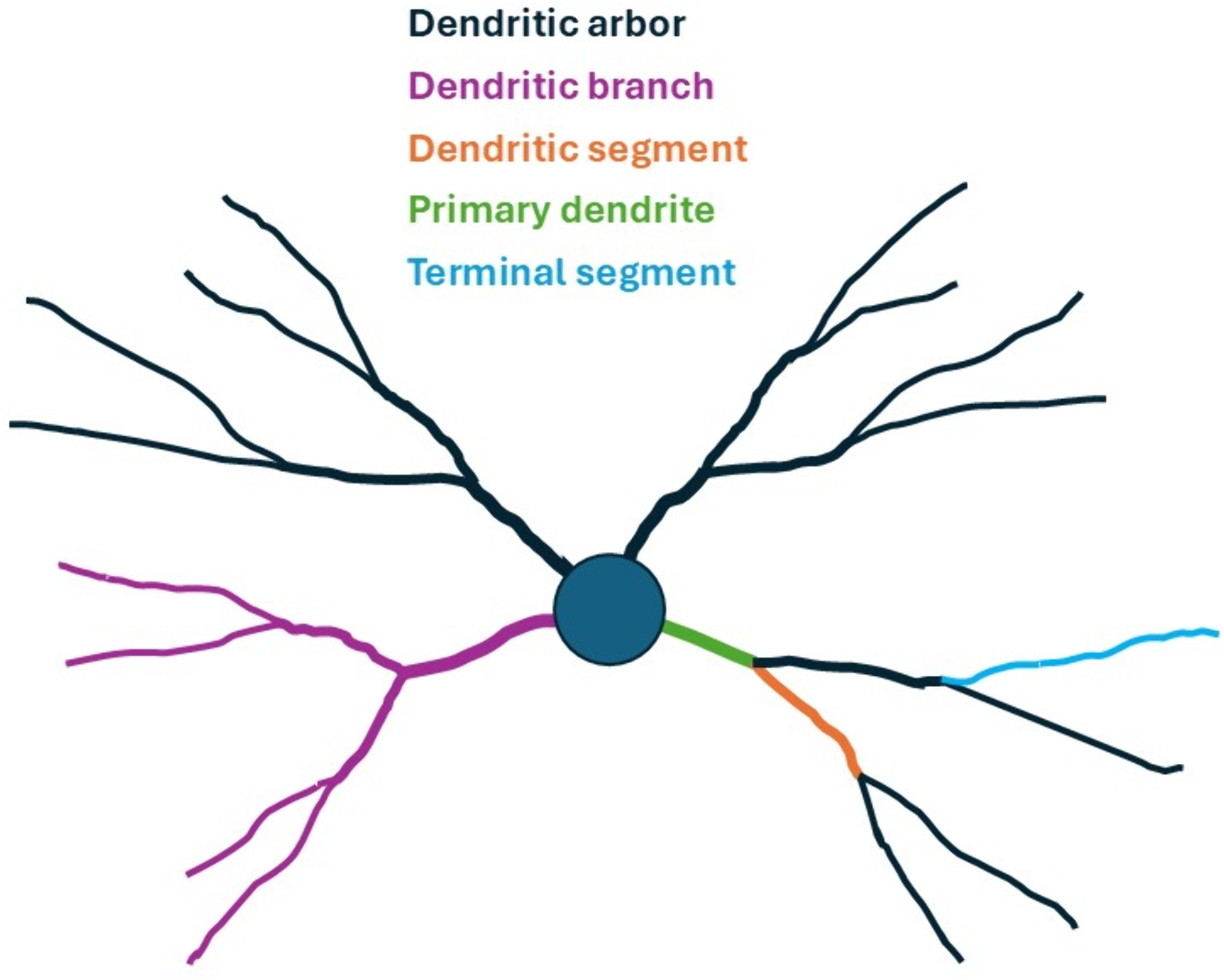

Supplement: S2 Fig — Nomenclature of dendritic morphometry. Dendritic arbor - the whole set of dendrites of the neuron. Dendritic branch - the subset of dendrites that extends directly from the soma. Dendritic segment - a portion of a dendrite between two structural points (e.g., two branching points, soma and a branching point or a tip). Primary dendrite - initial, main dendritic segment that originates at the soma. Terminal segments - the most distal segments of a dendritic branch, not undergoing further branching. Branch order - hierarchical level of a dendritic segment within the tree in centrifugal order; primary dendrite is assigned branch order 1, secondary segments have branch order 2 and so on, with higher numbers indicating more distal segments from the soma. Branch breadth - the total number of terminals of a given branch, defines the hierarchical level of a segment in centripetal order; the terminal segments have breadth 1, the next segments after the branching points in the direction towards the soma have breadth 2, and so on; breadth of a given segment equals the sum of the breadths of its daughter branches. (TIF) [file pcbi.1013569.s002.tif]

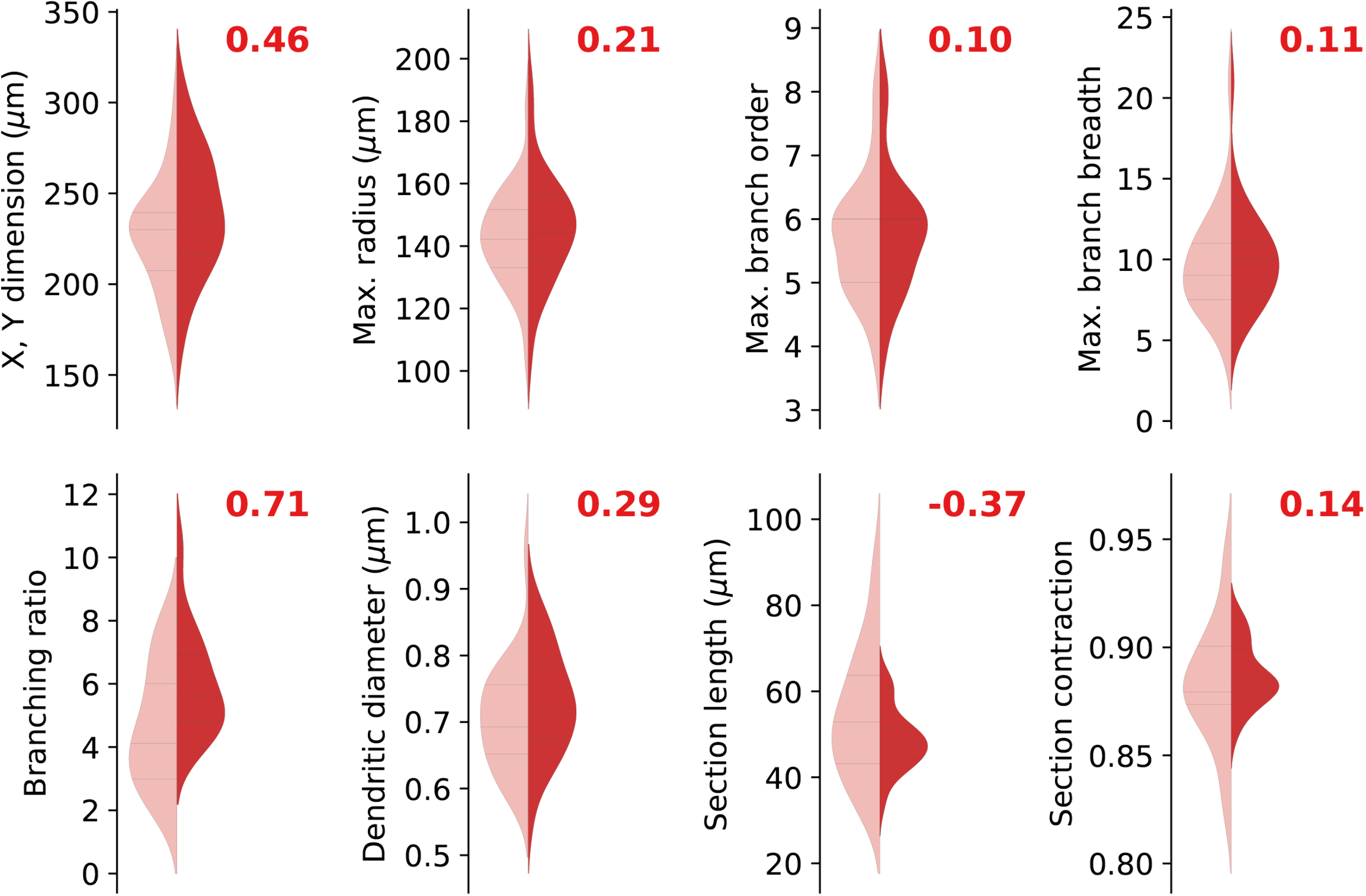

Supplement: S3 Fig — Validation of the repair process for mouse SPNs. Distributions of morphometric features of the repaired reconstructions (dark red, 165 dendrites in 31 reconstructions) is compared to morphometry of the complete dendrites (light red, 47 complete dendrites). Z-scores are shown in bold for each morphometric feature. (TIF) [file pcbi.1013569.s003.tif]

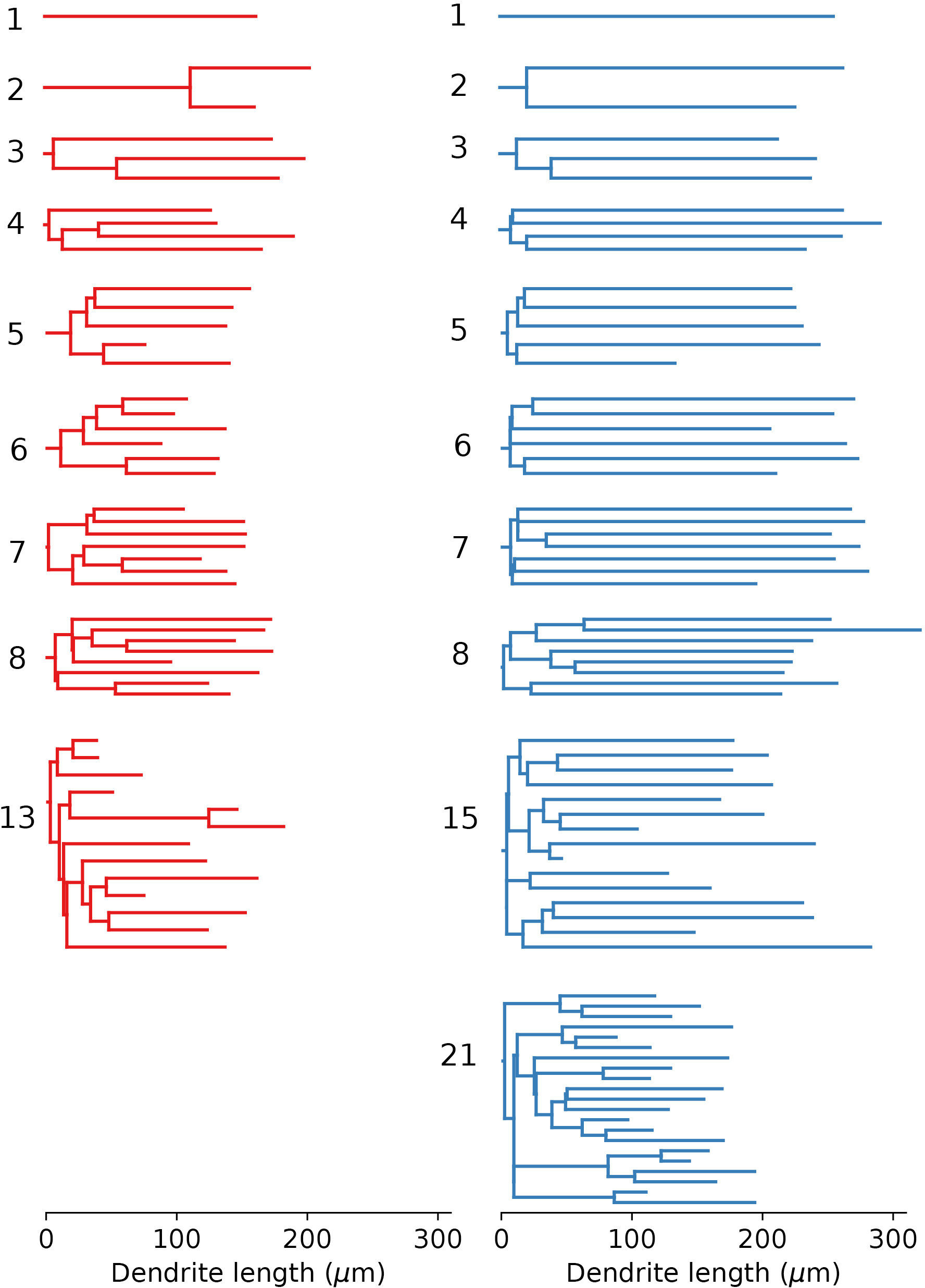

Supplement: S4 Fig — Dendrograms of the dendritic branches of different topological breadth (number of terminals). Red for mouse, blue for human, the branch breadth number is stated to the left of each dendrogram. Only one example dendrite is shown for each breadth. In the current dataset, the mouse dendrites have breadth 1 (n = 8), 2 (n = 4), 3 (n = 6), 4 (n = 9), 5 (n = 6), 6 (n = 4), 7 (n = 4), 8 (n = 4), and 13 (n = 1). Human dendrites have breadth 1 (n = 4), 2 (n = 4), 3 (n = 9), 4 (n = 6), 5 (n = 2), 6 (n = 5), 7 (n = 1), 8 (n = 2), 15 (n = 1), and 21 (n = 1). (TIF) [file pcbi.1013569.s004.tif]

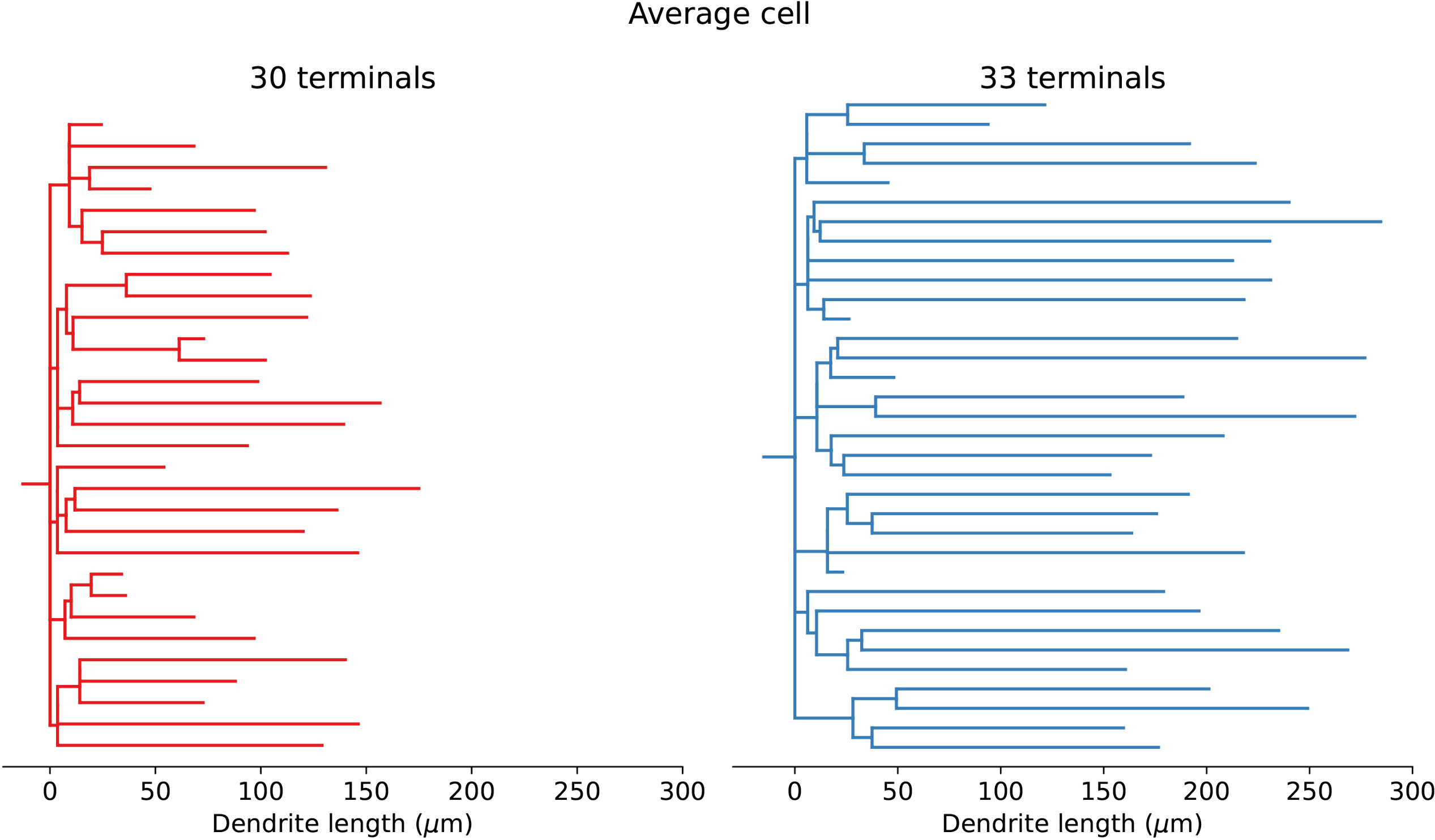

Supplement: S5 Fig — Dendrograms of the average repaired single-cell reconstructions. The repaired reconstructions of human SPNs are similar in shape and structure to those of the mouse with 5–6 primary dendrites (mean values 5.4 and 5.6 for mouse and human, respectively) and about 30 terminals per neuron on average (30.1 and 33.2). (TIF) [file pcbi.1013569.s005.tif]

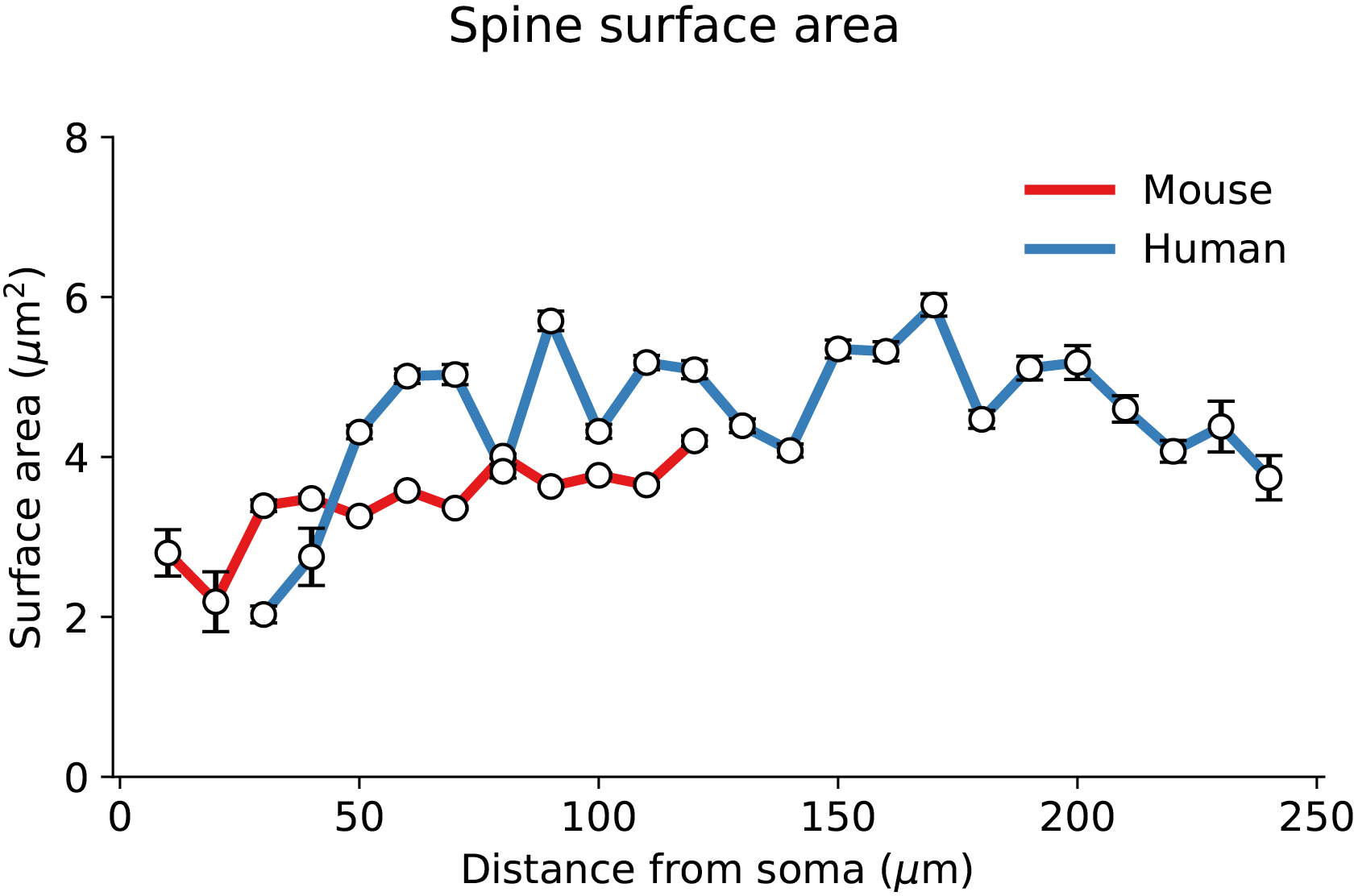

Supplement: S6 Fig — Surface area (μm2) of the dendritic spines as a function of distance from soma along the dendrite (mean and SEM) for the human and mouse neurons (see also S3 Table). (TIF) [file pcbi.1013569.s006.tif]

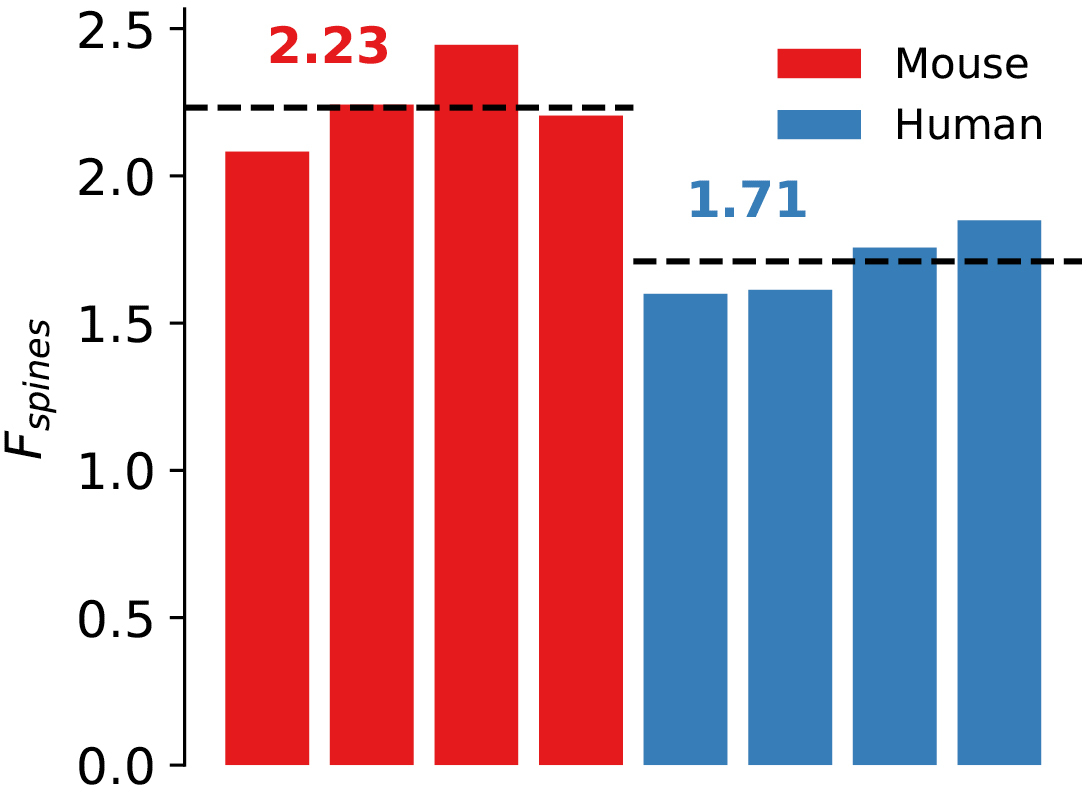

Supplement: S7 Fig — Factor Fspines=Aspines+AshaftAshaft estimated for four selected dendrites in mouse (2.23 ± 0.15) and human (1.71 ± 0.12) neurons. (TIF) [file pcbi.1013569.s007.tif]

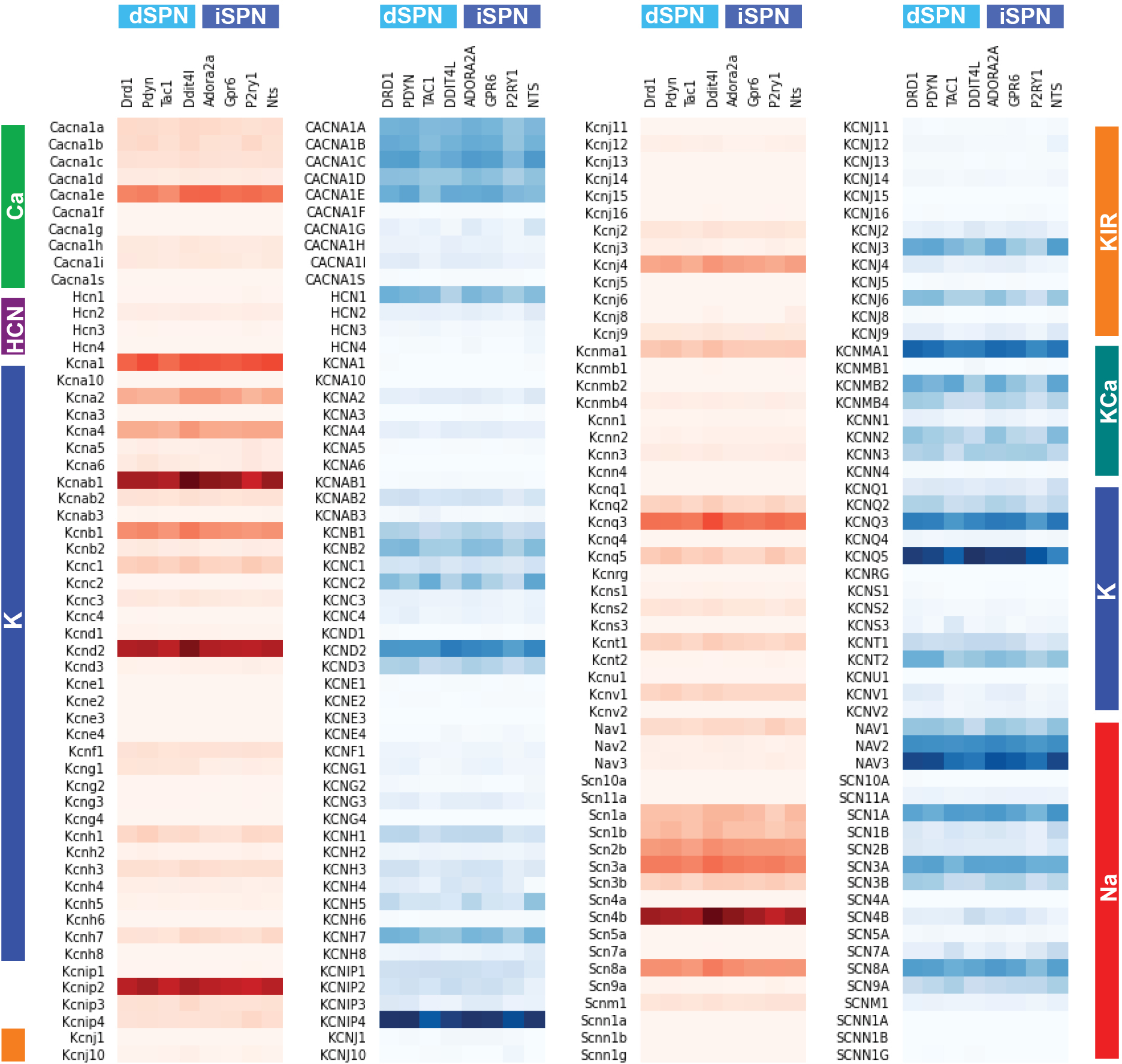

Supplement: S8 Fig — Comparison of the ion channel gene expression patterns in mouse and human SPNs (red and blue palettes, respectively). Data from Saunders et al. [30] for mice and Siletti et al. [31] for humans. (TIF) [file pcbi.1013569.s008.tif]

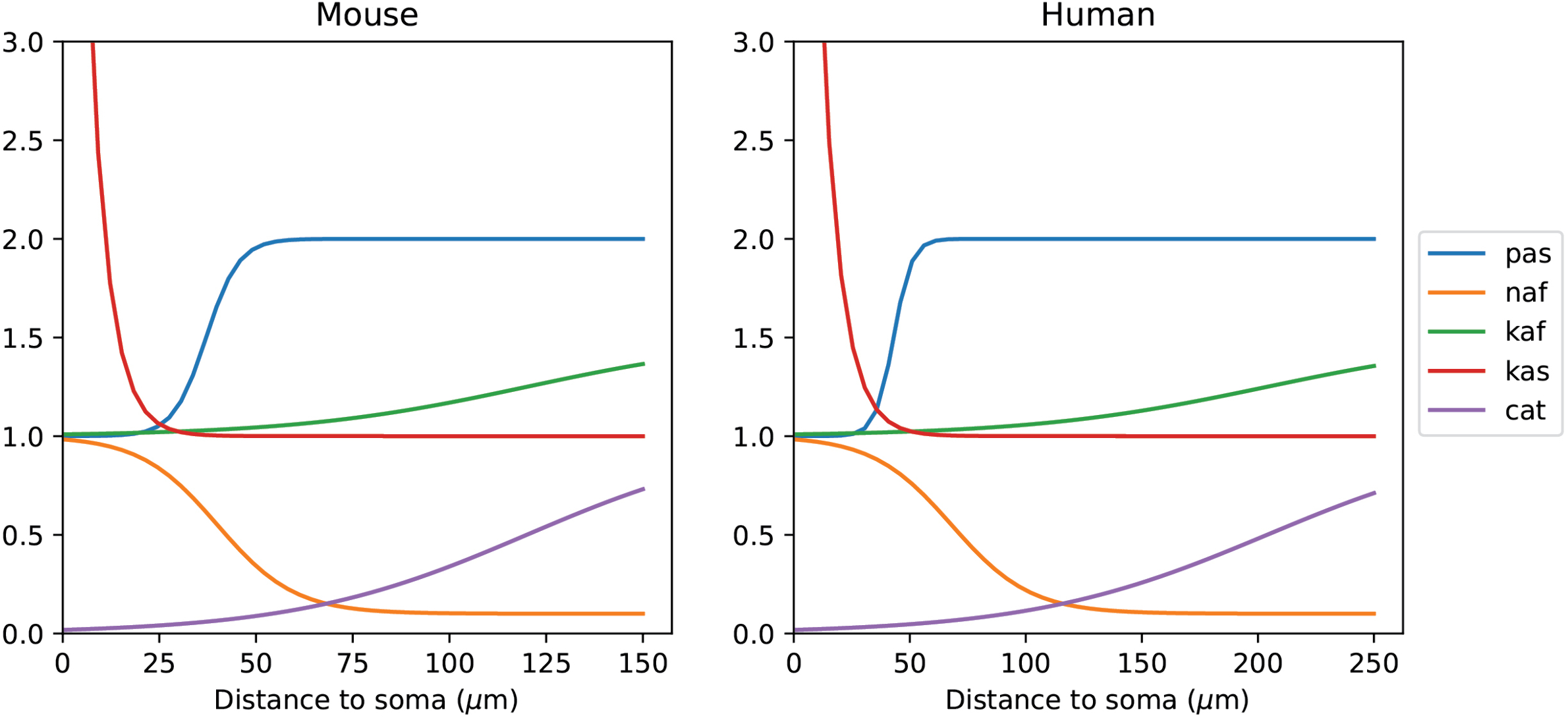

Supplement: S9 Fig — Distribution of electric conductances along the dendrites with distance from soma as in Hjorth et al. [33]. Here pas stands for the passive membrane conductance and membrane capacitance Cm, naf for the fast Nav ion channel, kaf and kas for the fast and slow components of A-type K+ channel, and cat for a T-type Ca2+ ion channel. Conductances of the active ion channels in the human model are extended by 70%. Distance dependence of passive conductance and membrane capacitance is fitted to the spine density distribution in mouse and human reconstructions. The Fspines factor to account for the membrane area of spines is set to 2.0 in both models. (TIF) [file pcbi.1013569.s009.tif]

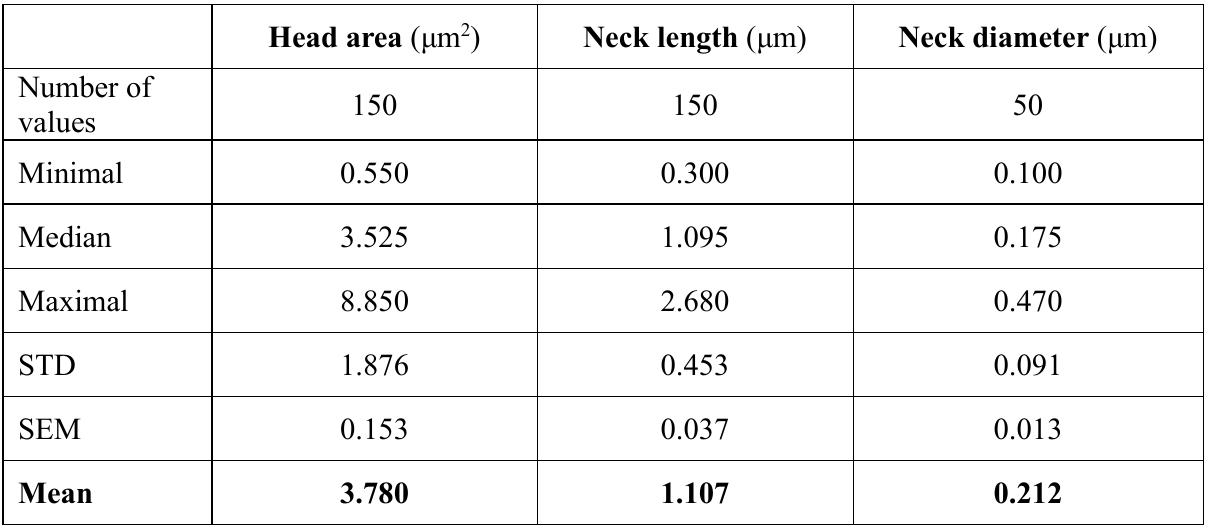

Supplement: S1 Table — Spine dimensions of the human striatal projection neurons (see Fig 6 for illustration). (TIF) [file pcbi.1013569.s010.tif]

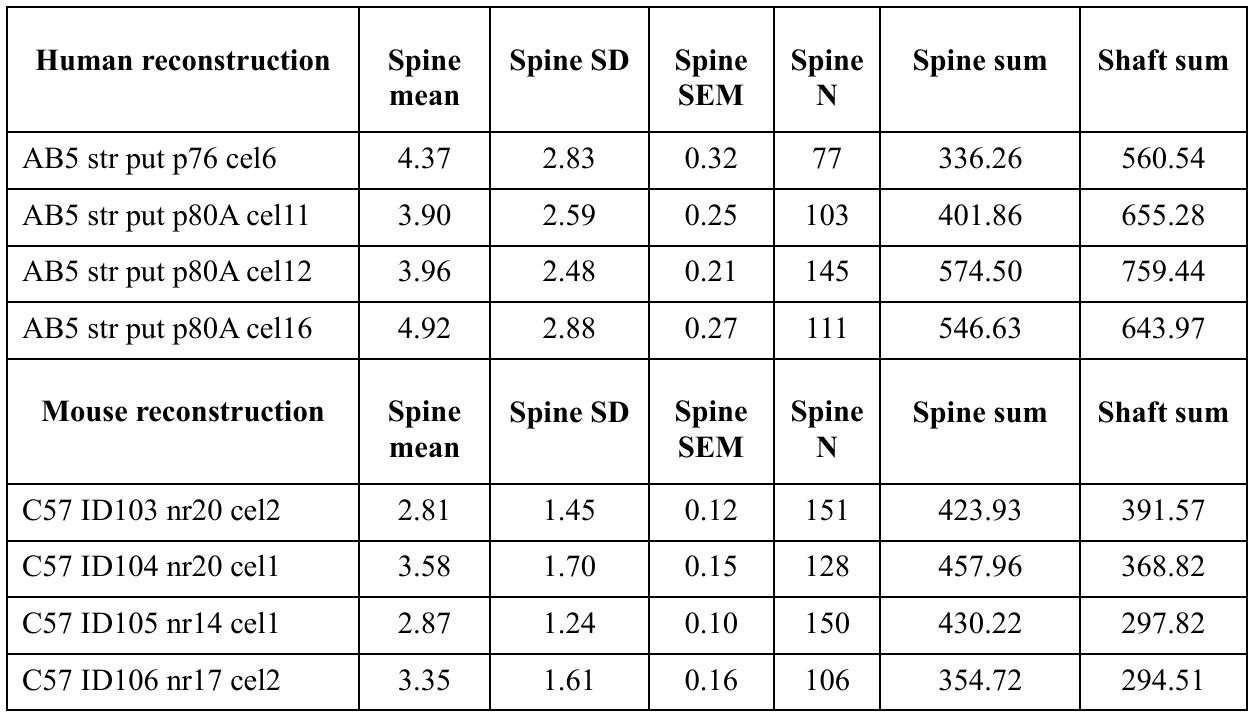

Supplement: S2 Table — Surface area (μm2) of the dendritic spines and the corresponding dendritic shaft in human and mouse dendrites, 4 dendrites each (see Fig 7). (TIF) [file pcbi.1013569.s011.tif]

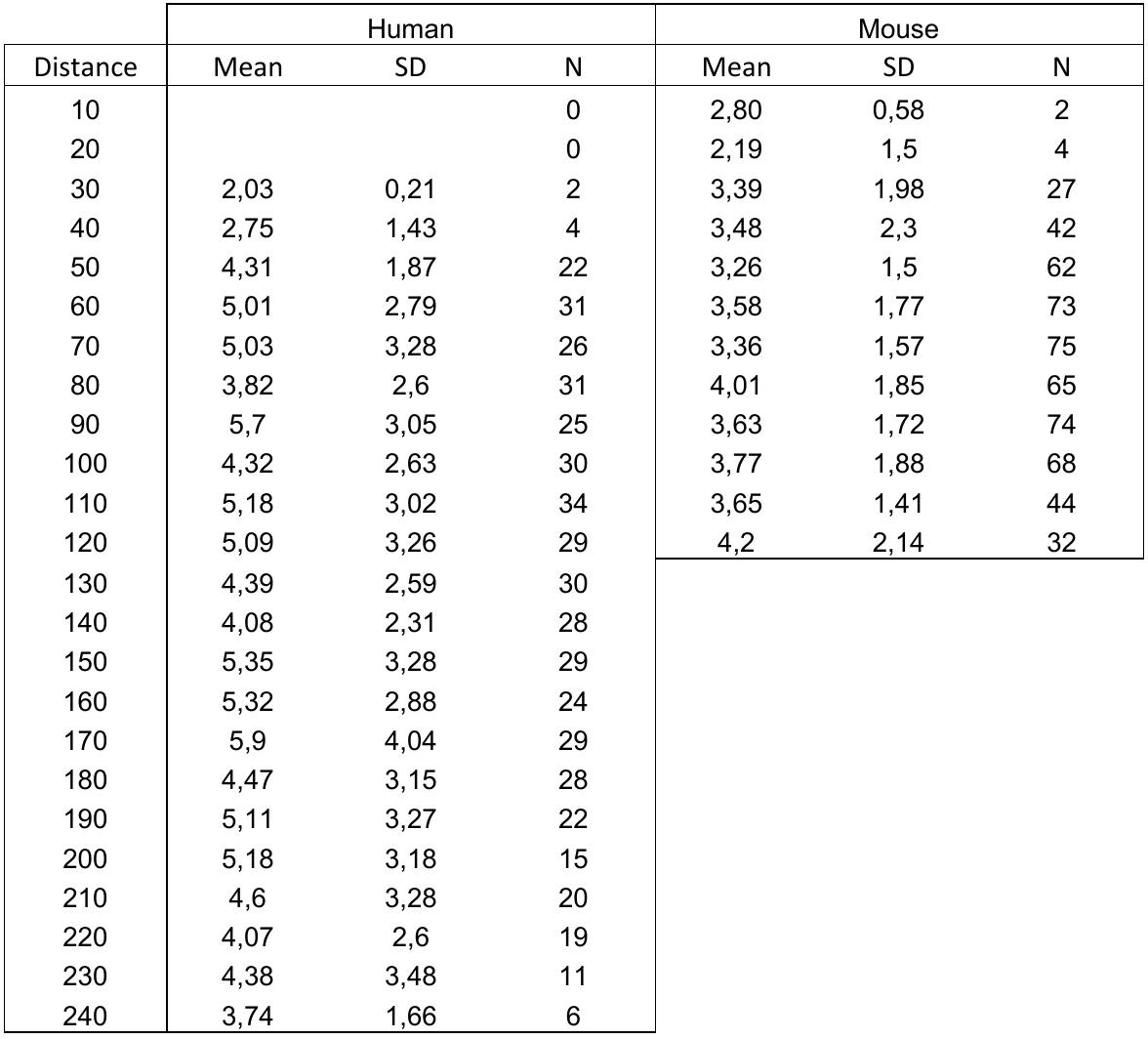

Supplement: S3 Table — Numerical values for S6 Fig on the dependence of the spine surface area (μm2) on the distance from soma (μm); mean and standard deviation, N is the number of spines reconstructed at a given distance from the soma. (TIF) [file pcbi.1013569.s012.tif]
